# Advancing Biotherapeutic Treatment of Ulcerative Colitis Using Lipid Mesophases: Formulation Design and Preclinical Validation

**DOI:** 10.1002/advs.78234

**Published:** 2026-10-11

**Authors:** Rafaela Gazzi, Tiziana Cremona, Reto Czekalla, Emily Campbell, Marlene Schwarzfischer, Doris Pöhlmann, Vanessa Dobernig, Gerhard Rogler, Michael Scharl, Alessandra Bergadano, Raffaele Mezzenga, Martin Kuentz, Paola Luciani, Simone Aleandri

**Affiliations:** ^1^ Department of Chemistry Biochemistry and Pharmaceutical Sciences University of Bern Bern Switzerland; ^2^ Department of Gastroenterology and Hepatology University Hospital Zurich University of Zurich Zurich Switzerland; ^3^ Experimental Animal Center (EAC) University of Bern Bern Switzerland; ^4^ Laboratory of Food & Soft Materials Institute of Food Nutrition and Health (IFNH) Department for Health Sciences and Technology (D‐HEST) ETH Zurich Zurich Switzerland; ^5^ Pharma Technology and Biotechnology University of Applied Sciences and Arts Northwestern Switzerland Muttenz Switzerland

**Keywords:** colon‐targeted drug delivery system, infliximab, lipid mesophases, localized delivery of biotherapeutics, ulcerative colitis

## Abstract

Biotherapeutics are required to achieve high remission rates in patients with severe ulcerative colitis (UC); however, adverse effects, complex dosing regimens, administration routes, and low patient compliance limit their widespread clinical use. Given the localized nature of UC, this study aimed to develop localized delivery strategies for infliximab (IFX), an anti‐tumor necrosis factor‐α (TNF‐α) monoclonal antibody recommended for moderately‐to‐severely active UC. Exploiting the intrinsic biocompatibility, mucoadhesion, and protein‐entrapment capacity of lipid mesophases (LMPs), IFX was encapsulated within the gel matrix, providing protection against enzymatic and environmental degradation. IFX‐LMPs were designed for targeted delivery to inflamed colonic tissues via rectal or oral administration, with oral dosage forms manufactured either by 3D printing or by encapsulation into enteric capsules. A comprehensive physicochemical characterization was performed to elucidate mesophase self‐assembly and its relationship with IFX release profiles in biorelevant fluids. The rectal gel demonstrated strong mucoadhesion, while LMP‐loaded enteric capsules enabled effective colon targeting. In a prophylactic treatment setting, LMP‐based formulations significantly attenuated disease progression and the development of severe inflammation compared with subcutaneous IFX. Overall, this study establishes a biocompatible delivery platform that enables colon‐targeted IFX release, representing a promising therapeutic strategy for UC using biotherapeutics.

## Graphical abstract

Lipid mesophases provide a versatile platform for colon‐targeted delivery of biotherapeutics, protecting them from degradation while enhancing mucoadhesion and enabling localized, controlled release to the inflamed mucosa after oral or rectal administration. In a DSS‐induced colitis model, this strategy reduced disease activity, inflammatory biomarkers, and histological damage, highlighting its therapeutic potential.

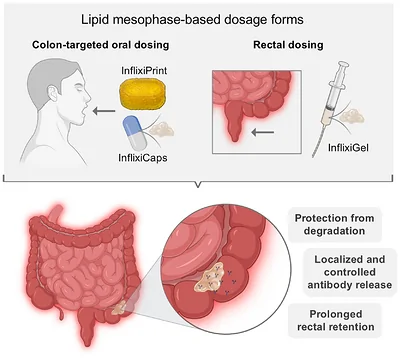

## 1 Introduction

Ulcerative colitis (UC) is a chronic inflammatory disorder of the colonic mucosa characterized by continuous inflammation and ulceration. Its pathogenesis involves a complex interplay between genetic and environmental factors [1, 2, 3, 4], leading to symptoms that severely impair patients’ quality of life [1, 2]. UC typically begins in the rectum (proctitis) and may progress proximally to involve the entire colon (pancolitis) [1, 2]. Disease location and severity guide treatment selection [1, 5], which includes salicylates, corticosteroids, calcineurin inhibitors, thiopurines, Janus kinase inhibitors and biotherapeutics such as monoclonal antibodies (mAbs) [1, 2, 6].

Biologic therapies are recommended within a step‐up approach for patients who fail to respond adequately to corticosteroids [7] and mainly include IFX (TNF‐α), vedolizumab (anti‐α4β7 integrin), and ustekinumab (anti‐IL‐12/IL‐23), all of which demonstrate comparable clinical outcomes [8, 9, 10, 11]. Among these, IFX remains one of the most prescribed medications and is recommended by ECCO guidelines for moderately‐to‐severely active UC [7]. IFX demonstrates superior efficacy in inducing clinical [12] and endoscopic remission [13] compared with other mAbs, and it is cost‐effective during the first two years following diagnosis [14]. Nevertheless, its clinical effectiveness may be compromised by immunogenicity, systemic toxicity, and treatment‐related adverse events [15, 16], with infusion‐related reactions being the most frequently reported [5]. Moreover, loss of response to biologics remains a major challenge, as up to 40% of patients in clinical remission develop anti‐drug antibodies during IFX therapy [5, 17]. Together with these limitations, the burden of repeated dosing and administration, requiring subcutaneous injections or intravenous infusions every 2 and 8 weeks, respectively, during maintenance therapy, may compromise patient adherence and consequently treatment effectiveness. Therefore, the therapeutic benefits of IFX must be balanced against its potential adverse effects.

Because UC is confined to the colon, targeted drug delivery strategies are particularly attractive as they enable high mAb concentrations at sites of inflammation, thereby minimizing exposure to healthy tissues and reducing immunogenicity [18]. Localized delivery can be achieved through rectal administration for distal UC (proctitis) or oral administration for extensive UC (pancolitis), potentially mitigating both anti‐drug antibody formation and infusion‐related reactions. However, colonic drug delivery poses route‐dependent formulation challenges: conventional rectal drug formulations often exhibit poor mucoadhesion and leakage, while oral formulations must protect the drug during gastric transit and enable colonic release. To address these challenges, bioadhesive rectal [19] and gastro‐resistant oral [20, 21] protein‐based formulations have been proposed.

Within this context, LMPs represent an attractive alternative drug delivery system, consisting of biodegradable gels capable of encapsulating hydrophilic, lipophilic, and amphiphilic molecules. One of their most prominent applications has been in the crystallization of membrane proteins [22]. Additionally, proteins can be immobilized within the mesophase matrix, preventing enzymatic and environmental degradation while enabling controlled release [23]. The safety and industrial translatability of LMPs are supported by the FluidCrystal technology developed by Camurus, a subcutaneous in situ‐forming LMP gel. Moreover, we have previously demonstrated the efficacy of monolinolein (MLO)‐based LMPs as a local treatment for UC [24]. Upon hydration, the self‐assembly of MLO is driven by the hydrophobic effect, lipid molecular structure, and water content, resulting in the formation of different liquid‐crystalline structures. With increasing water content, the flat lipid bilayers of the lamellar phase (L) transform into a curved, bicontinuous cubic phase (Q; with Ia3d, Pn3m or Im3m geometry), whose appearance and rheological properties resemble those of cross‐linked polymeric hydrogels [25]. These cubic mesophases have attracted particular attention due to their high surface area‐to‐volume ratio, high water uptake capacity, typically accounting for approximately 50% of the total volume, and complex internal topologies [26]. While this architecture is advantageous for accommodating and potentially stabilizing sensitive proteins, their narrow aqueous channels (approximately 3 nm) restrict the diffusion and release of large molecules such as mAbs [23, 27, 28]. Previous studies have addressed this limitation by enlarging water channels through hydration‐modulating agents [29, 30] or charged lipids [28, 31, 32, 33].

In this study, we incorporated the hydration‐modulating agent sucrose stearate (SE) into the mesophase composition to engineer localized mAb delivery systems. It has been demonstrated that the incorporation of SE into MLO‐based LMPs reduces the curvature of the lipid bilayer toward the aqueous phase, thereby promoting the transition from the Pn3m to the Im3m cubic phase and increasing the diameter of the aqueous channels [29]. Beyond its swelling properties, SE modulates LMP erosion in colonic fluids and reduces gel rigidity, thereby facilitating formulation handling. The resulting SE‐doped gel exhibited optimal rheology for rectal administration and for semisolid extrusion (SSE) 3D printing of an oral dosage form, consisting of a gastro‐resistant shell [34] and a mAb‐loaded core with release triggered by intestinal fluids. With this formulation approach, we propose a single starting material that can be used for two administration routes, with the appropriate dosage form selected according to disease extent. Employing LMPs as a starting material to encapsulate mAbs and using this material as a bioink for 3D‐printed oral dosage forms provides a breakthrough platform for oral biotherapeutic delivery. The LMP matrix can protect mAbs from degradation, while 3D printing enables the fabrication of complex geometries with precisely defined wall thicknesses and multilayered compartments, providing control over the rate and location of drug release along the gastrointestinal tract.

Oral biotherapeutic delivery has been widely explored to minimize the adverse effects of systemic treatments. Recent strategies rely on nanocarriers formulated as solutions, administered either as liquid formulations [20, 21] or capsules [35]. However, these approaches remain limited by formulation stability and challenges in clinical translation. Therefore, to streamline manufacturing while preserving mAb stability, we propose a biocompatible, mucoadhesive LMP‐based platform for colon‐targeted IFX delivery. The IFX‐loaded LMP matrix was formulated for rectal administration as a gel (InflixiGel) and for oral administration either as enteric capsules (InflixiCaps) or as 3D‐printed dosage forms, using the LMP as bioink (InflixiPrint). The therapeutic potential of the LMP‐based formulations was evaluated in vivo using InflixiGel and InflixiCaps in a DSS‐induced colitis model in rats, where they significantly attenuated disease manifestations. To the best of our knowledge, the use of LMPs for mAb delivery, and particularly their integration with additive manufacturing to produce oral dosage forms, has not yet been explored. This combination provides a potential route toward on‐demand manufacturing of personalized oral formulations. Overall, these localized therapeutic approaches offer a versatile, patient‐centric, and potentially translational platform for biotherapeutic delivery.

## 2 Results

### 2.1 Formulation Development and Physicochemical Characterization

To reduce IFX‐related adverse effects while maximizing local drug exposure, we developed and characterized LMP‐based localized therapies designed to enable controlled and localized mAb release directly at the inflamed intestinal mucosa. Because the extent and location of inflammation in UC can vary along the colon, we investigated a single starting material for the development of both rectal and oral formulations, designed to address different disease localizations and delivery constraints. Therefore, when inflammation is limited to the distal colon (e.g., proctitis), the rectal formulation InflixiGel could be selected, whereas for more extensive colonic involvement, the oral formulations InflixiPrint and InflixiCaps could provide broader colonic coverage. Oral dosage forms can enable colon‐specific drug delivery by using coatings responsive to specific gastrointestinal (GI) conditions. InflixiPrint has an LMP‐based gastro‐resistant layer that dissolves in the intestine, allowing the IFX‐loaded LMP in the core to swell and erode, triggering mAb release. This gastro‐resistant shell was composed of the phospholipid S80 and vitamin E (S80/VitE_LMP; Figure 1A), whose composition and printing parameters were previously validated [34]. The core consisted of the lipid MLO and the hydration‐modulating agent SE (MLO/SE_LMP; Figure 1B), and its optimal composition for printability was determined by varying the MLO‐to‐SE ratio (Figure 1C). Hydration was set at 50% w/w for all MLO/SE LMPs, except for the formulation without SE, where a maximum hydration level was reached with 40% w/w of water [36].

**FIGURE 1:**
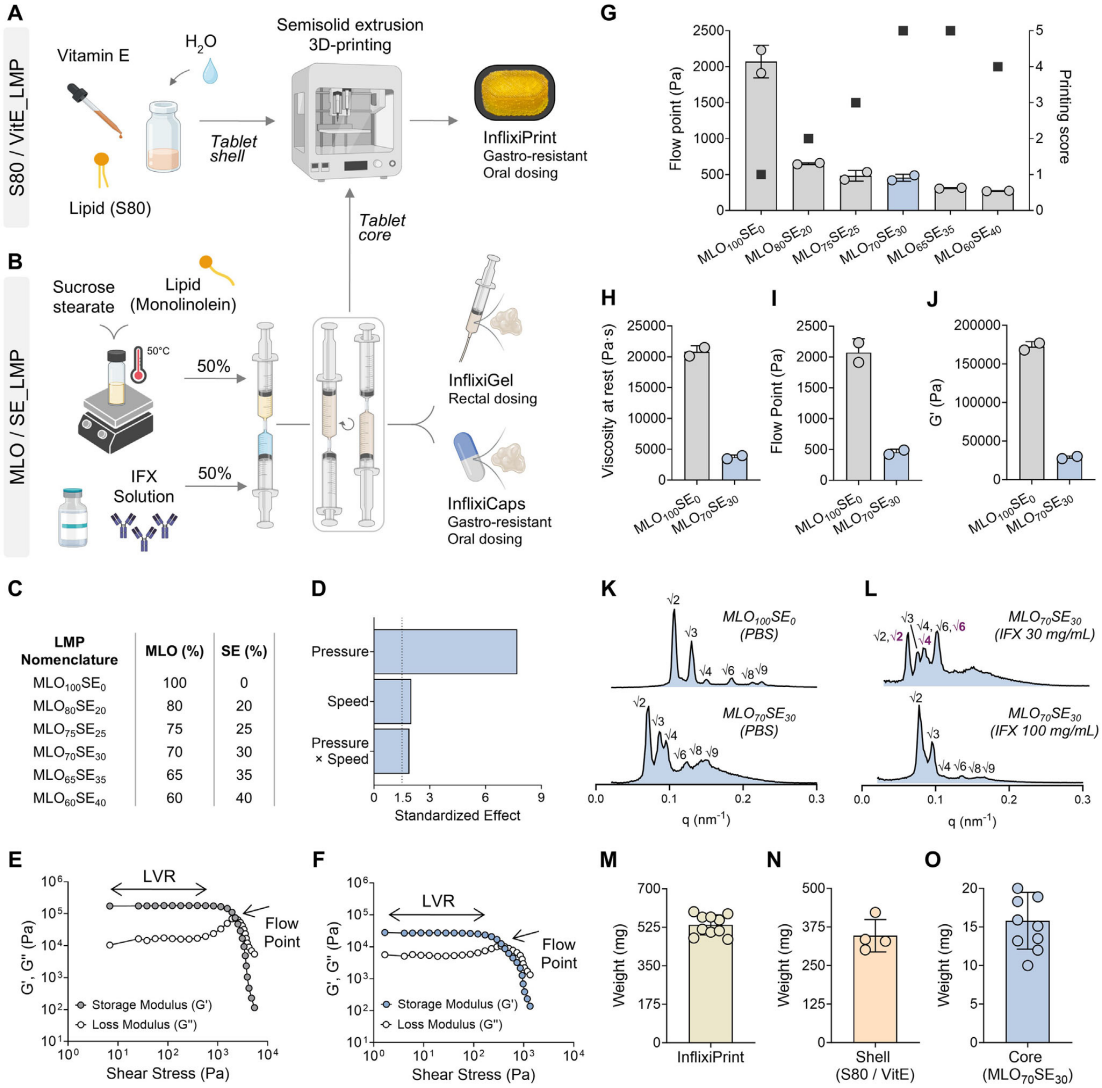
Production and physicochemical characterization of LMPs. (A) Development of S80/VitE_LMP and production of InflixiPrint. (B) Development of MLO/SE_LMP and production of InflixiGel and InflixiCaps. (C) Range of monolinolein (MLO) and sucrose stearate (SE) tested for 3D printing MLO/SE_LMPs. (D) Pareto chart of MLO_70_SE_30_. Amplitude sweeps of (E) MLO_100_SE_0_ and (F) MLO_70_SE_30_. Linear viscoelastic region (LVR) and flow points are indicated by arrows. (G) Correlation between flow point (bars) and maximum printing score (squares) for LMPs formulated with lipid mixtures containing 0%–40% SE. (H) Viscosity at rest, (I) flow point, and (J) G′ at the LVR of MLO_100_SE_0_ and MLO_70_SE_30_. (K) Phase identity of MLO_100_SE_0_ and MLO_70_SE_30_ hydrated with PBS and (L) phase identity of MLO_70_SE_30_ hydrated with IFX solution at 30 and 100 mg/mL. The Bragg reflections for the cubic Pn3m and Im3m phases are reported above each peak in black and purple, respectively. Average weights of (M) InflixiPrint, (N) S80/VitE shell, and (O) MLO_70_SE_30_ core. In panels G, H, I, J, M, N, and O, results are shown as single point values ± SD.

Given the high viscosity and heat sensitivity of lipids and IFX, we employed SSE 3D printing to produce oral dosage forms. The optimal printing parameters for each ink composition were identified using a full factorial Design of Experiments (DoE; Table S1). For each run, a printability score (1 = lowest, 5 = highest) was assigned (Figure S1), with these values being used to model the influence of the experimental factors and their interactions on printability. All main effects and interactions were evaluated. Factors that did not show statistically significant effects (*p* ≤ 0.10) were excluded from the final model. The formulation without SE exhibited poor printability, with the ink successfully extruded in only three of the 36 runs (Table S1). Consequently, the data were insufficient for analysis, and the DoE results for this formulation were not reported. The main effects and interaction plots for the remaining formulations are shown in Figure S2, while the Pareto charts show the magnitude of each effect on printability (Figure 1D for MLO_70_SE_30,_ and Figure S3 for other SE percentages). Pressure had the largest main effect on the printability score across all formulations. Moreover, printing trials showed that higher sugar content reduced the pressure required for ink extrusion. Thus, to further elucidate the rheological behavior of the inks, viscosity‐shear rate curves and amplitude sweep tests were performed; the latter was used to determine the storage modulus (G′) within the linear viscoelastic region (LVR) and flow points (Figure 1E,F). Figure 1G shows the correlation between the flow point and the highest printability score obtained for each formulation. The highest printability score [5] was obtained for MLO_70_SE_30_ and MLO_65_SE_35_ (Figure 1G); however, MLO_70_SE_30_ was selected because fewer factors needed to be optimized for printing, as shown in Figure S2. The flow point of MLO_100_SE_0_ was 4.4‐fold higher than the average of the other formulations (Figure 1G), explaining its poor printability and highlighting the importance of SE in facilitating extrusion. As shown in Figure 1H–J, the viscosity at rest, flow point and G’ for MLO_100_SE_0_ were 5.6‐fold, 3.9‐fold and 5.9‐fold higher than those of the lead candidate MLO_70_SE_30_, respectively, further supporting this observation.

The influence of SE and IFX on the phase identity of MLO‐based LMPs was investigated using SAXS. The first spectrum in Figure 1K (MLO_100_SE_0_) shows Bragg peaks with positions in the ratio √2: √3: √4: √6: √8: √9, characteristic of the Pn3m inverse bicontinuous cubic phase. The addition of 30% SE in the lipid phase did not alter mesophase identity (Figure 1K, second spectrum). However, the shift of Bragg peaks to lower *q*‐values indicates increased lattice parameter and larger water channels. The swelling effect was further confirmed by the greater water uptake capacity of formulations containing SE (Figure S4). SE percentages above 40% led to amorphous gels with unknown crystalline domains (Figure S5). Hydration of MLO_70_SE_30_ with a 30 mg/mL IFX solution shifted the phase boundaries of the system, resulting in the coexistence of Pn3m and Im3m symmetries and a further shift of the Bragg peaks to lower *q*‐values (Figure 1L, first spectrum). At a higher IFX concentration (100 mg/mL), the mesophase shrank, reducing its lattice parameter and water channel dimensions (Figure 1L, second spectrum).

The optimal printing parameters identified by the DoE for MLO_70_SE_30_ were a pressure of 110 kPa, a printing speed of 3 mm/s, and an extrusion temperature of 35°C, resulting in 3D‐printed tablets of reproducible size and weight (Figure 1M–O; Figure S6A). For comparison with the 3D‐printed tablets (InflixiPrint; Figure 1A), capsules were filled with MLO_70_SE_30_ and coated with Eudragit FS30D to promote colon targeting (InflixiCaps; Figure 1B). The LMP gel was hydrated with a 100 mg/mL IFX solution, delivering 7 mg of IFX per tablet/capsule. The rectal formulation (InflixiGel; Figure 1B) was hydrated with a 30 mg/mL IFX solution and delivered at a dose of 1.5 mg IFX per 100 mg of gel. The secondary structure of IFX in the solutions used to hydrate the gels was assessed by circular dichroism across the full concentration range investigated (30, 100, and 180 mg/mL; Figure S7). The spectra demonstrated preservation of the characteristic β‐sheet secondary structure of the antibody at all tested concentrations, indicating that no apparent changes in protein conformation or aggregation occurred prior to formulation.

Collectively, these results demonstrate that formulation architecture, manufacturing strategy (SSE 3D printing vs. encapsulation and coating), and compositional tuning via SE incorporation modulate rheological behavior, mesophase structure, processability, and suitability for localized IFX delivery.

### 2.2 IFX Is Efficiently Encapsulated and Released From InflixiGel, InflixiPrint and InflixiCaps

IFX was efficiently encapsulated within the LMP‐based formulations, each exhibiting a formulation‐specific release profile. The gastro‐resistance of InflixiPrint was confirmed by the preservation of tablet integrity and the absence of detectable IFX or degradation products after incubation under acidic conditions (Figure 2A). Upon transferring to PBS, the gastro‐resistant shell of InflixiPrint remained largely intact for up to 75 min, preventing core exposure and resulting in no detectable IFX release after 6 h (Figure 2A,B). In contrast, shell disintegration occurred more rapidly in biorelevant intestinal fluids. In fasted‐state simulated intestinal fluid (FaSSIF), near‐complete shell disintegration occurred within 45 min, with partial core erosion observed at 75 min. In fed‐state simulated intestinal fluid (FeSSIF), both shell and core disintegrated faster, leading to complete tablet disintegration within 75 min (Figure 2A). Accordingly, IFX release after 6 h was higher in FeSSIF (67%) than in FaSSIF (29%) (Figure 2B).

**FIGURE 2:**
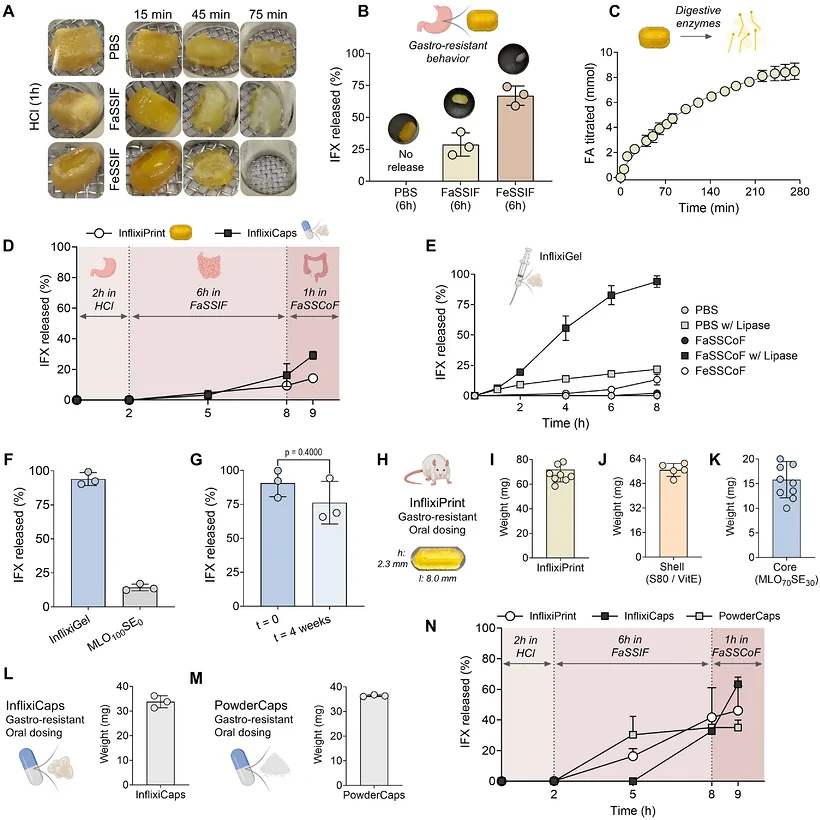
Behavior of formulations in simulated digestive fluids. (A) Disintegration of InflixiPrint in HCl followed by PBS, FaSSIF or FeSSIF. (B) Dissolution of InflixiPrint in PBS, FaSSIF, and FeSSIF. (C) Free fatty acids (FA) originating from InflixiPrint digestion as a function of time (results are shown as mean ± SD; *n* = 2). (D) IFX release from InflixiPrint and InflixiCaps as a function of time in HCl, FaSSIF, and FaSSCoF (results are shown as mean ± SD; *n* = 3). (E) IFX release from InflixiGel in PBS and simulated colonic fluids with or without lipase (results are shown as mean ± SD; *n* = 3). (F) Eight‐hour IFX release from InflixiGel or MLO_100_SE_0_ in FaSSCoF enriched with lipase. (G) Eight‐hour IFX release in FaSSCoF enriched with lipase from InflixiGel freshly prepared (*t* = 0) and after storage (*t* = 4 weeks). Miniaturized InflixiPrint (H) dimensions and (I) average weight, and the average weight of its (J) shell and (K) core. Average weights of miniaturized (L) InflixiCaps and (M) PowderCaps. In panels B, F, G, I, J, K, L, and M, results are shown as single point values ± SD. (N) IFX release from miniaturized oral dosage forms in HCl, FaSSIF, and FaSSCoF (results are shown as mean ± SD; *n* = 3). Statistical significance in G was evaluated by the nonparametric Mann–Whitney test. Adjusted *p*‐values are reported above the comparison.

Lipolysis studies under fasted‐state conditions showed progressive digestion of InflixiPrint, with approximately 8.5 mmol of fatty acids (FA) liberated over 240 min (Figure 2C). When the shell and core components were evaluated separately, lipolysis occurred faster, reaching completion within 90 min and releasing 6.5 and 3.2 mmol of FA from the shell and core, respectively (Figure S8). While these results qualitatively support the disintegration and dissolution data, direct comparison between experiments is not appropriate due to differences in experimental conditions and the absence of digestive enzymes in the disintegration and dissolution assays. The release profiles of InflixiPrint and enteric‐coated capsules (InflixiCaps) were compared (Figure 2D), and gastro‐resistance was confirmed for both formulations (Figure S9). In FaSSIF, InflixiPrint and InflixiCaps exhibited similar release profiles, releasing 9.5% and 16% of IFX, respectively, after 6 h. However, in fasted state simulated colonic fluid (FaSSCoF), IFX release from InflixiCaps was faster (29%) than from InflixiPrint (14%).

The release of IFX from InflixiGel was strongly medium‐dependent. Drug release was evaluated in PBS, FaSSCoF and fed‐state simulated colonic fluid (FeSSCoF), both with and without lipase (5000 U/mL) to mimic the presence of this enzyme in the inflamed colonic mucosa [37, 38] (Figure 2E). The rectal dosage form consisted solely of MLO_70_SE_30_ and, despite the absence of the S80/VitE shell, no IFX release was detected over 8 h in PBS. In simulated colonic fluids, IFX release remained limited (2% in FaSSCoF and 13% in FeSSCoF), with higher release in FeSSCoF attributed to its greater bile salt content. Lipase addition significantly increased IFX release in PBS and FaSSCoF (22% and 94%, respectively), indicating that release is primarily driven by enzymatic erosion of the LMP rather than passive diffusion through aqueous channels. While diffusion‐driven release can be described using mathematical models, the erosive component is inherently more difficult to model because it depends strongly on local enzyme activity and bile salt concentrations, both of which are subject to considerable inter‐individual variability. Given the experimental conditions of this study, mathematical modelling was therefore not applied, as it would not provide a reliable prediction of release behavior or a meaningful basis for establishing an in vitro–in vivo correlation. Gel erosion and drug release were not observed in lipase‐enriched FeSSCoF, likely due to enzyme inactivation by glucose present in this medium but absent in FaSSCoF [39].

To further investigate the role of the swelling agent, we compared IFX release from InflixiGel (MLO_70_SE_30_) and from a gel without SE (MLO_100_SE_0_). InflixiGel released nearly 100% IFX, whereas the release from MLO_100_SE_0_ was limited to 14% (Figure 2F), confirming the critical role of the SE in promoting drug release. This feature can be explained by the high hydrophilic–lipophilic balance value of the SE, which functions as a solubilizing agent for the LMP and facilitates its erosion. Formulation stability was assessed by comparing IFX release from freshly prepared InflixiGel with that from the gel stored at 4°C for four weeks, and comparable release profiles were observed at both time points (Figure 2G). Importantly, the amount released corresponded to biologically active IFX, as measured by ELISA. Therefore, these results indicate that IFX remained stable within the mesophase during four weeks of storage, with no apparent loss of its biological activity.

For in vivo evaluation, the InflixiPrint design was scaled down to match the dimensions of rat capsules (Torpac, size 9) (Figure 2H; Figure S6B). The weights of the core, shell, and total tablet were consistent across replicates (Figure 2I–K). For comparison, enteric‐coated capsules were filled with the same gel used to print the core of the 3D‐printed tablet (InflixiCaps; Figure 2L). We also investigated whether the LMP matrix was necessary for the oral delivery of IFX by filling colon‐targeting capsules with a wet powder mixture of mannitol and IFX solution (PowderCaps; Figure 2M). Despite their different compositions, InflixiCaps and PowderCaps exhibited comparable weights (Figure 2L,M). Miniaturized oral dosage forms were designed to deliver a dose of 2 mg/kg.

IFX release from the rat‐sized oral dosage forms was assessed under gastric and fasted intestinal conditions and compared with the release profile from human‐sized printlets and capsules (Figure 2N). Gastro‐resistance was confirmed by the absence of detectable IFX or degradation products after incubation in acidic conditions. For InflixiPrint, the change in medium composition from FaSSIF to FaSSCoF did not markedly affect the release rate, which is consistent with the observations for the human‐sized printlet. In contrast, InflixiCaps exhibited a burst release upon transfer to FaSSCoF, releasing approximately 30% of IFX within 1 h. PowderCaps exhibited faster release in FaSSIF, releasing 30% IFX within 3 h; however, no further release occurred in FaSSCoF. Although the coating of the capsules did not remain intact for the full 6 h in FaSSIF, its thickness was limited to ensure capsule compatibility with the gavage applicator. The maximum number of coating layers was applied while maintaining suitability for in vivo administration.

### 2.3 Gastrointestinal Transit of InflixiCaps and PowderCaps and Colonic Retention of InflixiGel

A pilot study was conducted to evaluate the feasibility of oral gavage of InflixiPrint and capsule formulations in rats. Although the 3D‐printed dosage form was sufficiently robust for human handling, its lipid‐based composition resulted in adhesion to the small gavage applicator and to the animals’ esophagus. This made administration difficult and uncomfortable, at least in small rodents like rats. Therefore, to prioritize animal welfare and to fulfil the 3R principles, InflixiPrint was excluded from the in vivo studies, and the GI transit was evaluated using InflixiCaps and PowderCaps. This represents a limitation of the present study, and further investigations in larger animal models, such as pigs, will be required to assess the translational potential and in vivo performance of the 3D‐printed patient‐centric oral formulation. Following oral gavage of barium sulfate‐loaded capsules, computed tomography (CT) imaging of the anesthetized rats was performed at predefined time points. Imaging at *t* = 0 confirmed gastric localization of both formulations immediately after administration (Figure 3A, top). At 4 h post‐gavage, InflixiCaps had progressed to the cecum, and they were consistently detected in the colon by 6 h (Figure 3A, left). In contrast, PowderCaps remained in the stomach at both 4 and 6 h (Figure 3A, right). These observations were reproducible across all animals (*n *= 3/group; Figure 3B), demonstrating that the LMP matrix facilitated efficient GI transit and timely colonic arrival.

**FIGURE 3:**
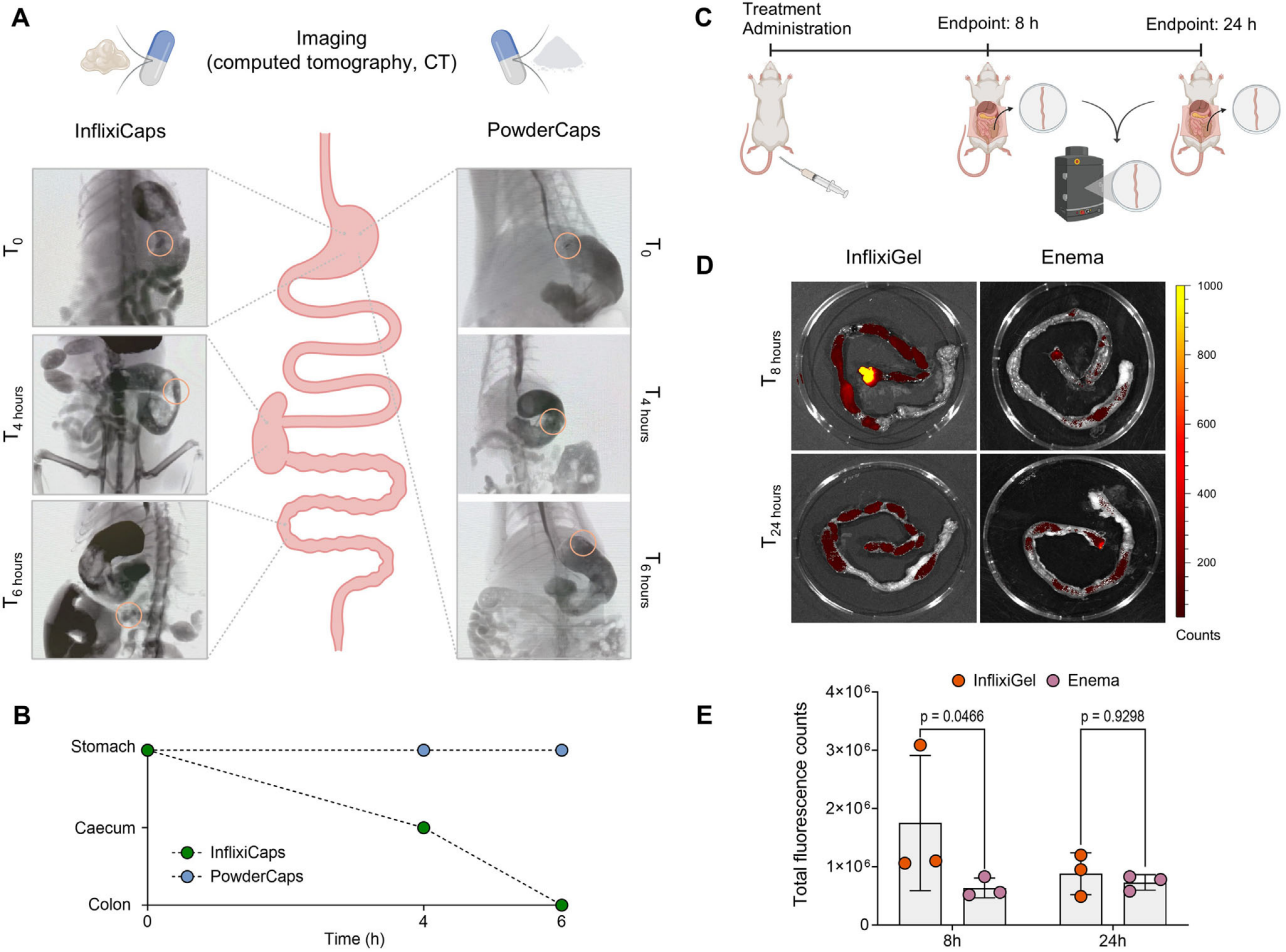
GI transit of oral formulations and colonic retention of rectal formulations. (A) Representative CT images of the rat GI tract over time after gavage of InflixiCaps and PowderCaps. (B) Location of InflixiCaps and PowderCaps in the GI tract over time (results are shown as mean ± SD; *n* = 3). (C) Experimental setup for assessing colonic mucoadhesion of rectal formulations. (D) Representative images of colons and (E) total fluorescence counts at 8 and 24 h after administration of rectal formulations (results are shown as single point values ± SD; *n* = 3). Statistical significance was evaluated by two‐way ANOVA with Tukey's post hoc analysis. Adjusted *p*‐values are reported above the comparison.

Insufficient mucoadhesion and leakage can compromise the efficacy of rectal formulations. Rectal formulations, such as enemas and suppositories containing mesalamine or corticosteroids, are commercially available and are generally recommended for the treatment of mild‐to‐moderate UC. In contrast, no topical rectal formulations of biotherapeutics are currently available. Therefore, we evaluated the colonic residence time of InflixiGel in comparison with an IFX enema, formulated as a buffered aqueous solution at the same drug concentration as in the gel. For reference, the viscosity of commercially available enemas typically ranges from 2 to 6.2 mPa s [40], while standard reconstituted IFX solutions have a viscosity of 1.5–3 mPa s at room temperature [41]. Fluorescently labelled rectal formulations were administered, and the rat colons were harvested 8 and 24 h post‐administration for fluorescence intensity analysis (Figure 3C). Representative images are shown in Figure 3D. After 8 h, the fluorescence signal in colons from InflixiGel‐treated rats was higher than that in the enema group, indicating stronger mucosal adhesion. After 24 h, the fluorescence signal in the InflixiGel‐treated group decreased approximately twofold and reached values comparable to those observed in the enema group (Figure 3E). Notably, the signal of the gel‐treated colons was higher than that of the untreated controls (Figure S10), confirming the sustained colonic presence of InflixiGel.

### 2.4 IFX Systemic Absorption and Colonic Retention

Systemic absorption and colonic retention of IFX were first characterized in DSS‐treated rats following subcutaneous administration (2 mg/kg), providing a systemic reference for our localized delivery systems. IFX reached a *C*_max_ of 23 µg/mL at 4 days post‐injection, with an AUC of 230 µg/mL × days over 22 days (Figure 4A–C). Colonic IFX levels increased 3.8‐fold by day 7 (0.16 µg/g tissue) and remained stable thereafter (Figure 4D).

**FIGURE 4:**
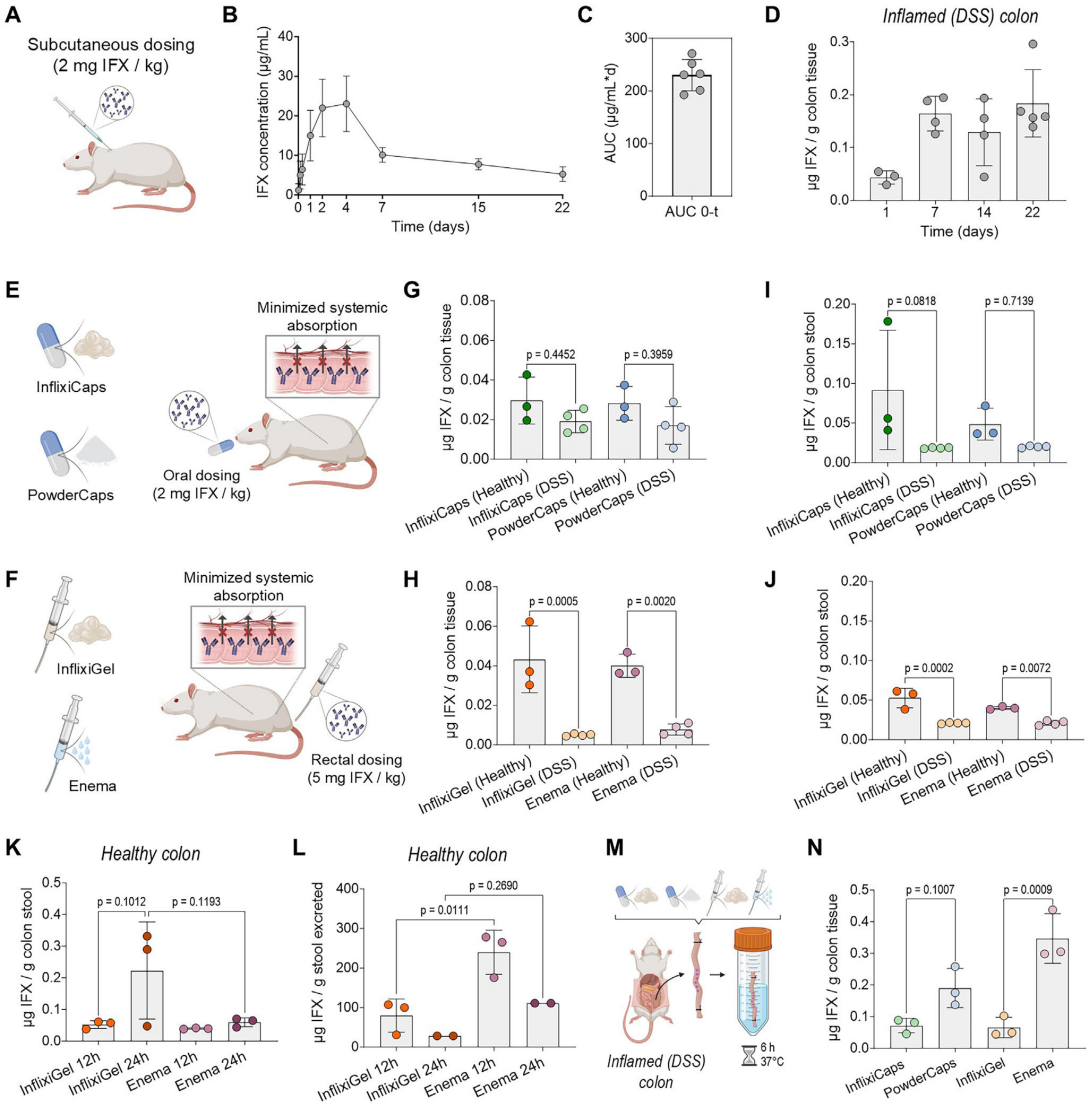
Blood and tissue pharmacokinetics after IFX subcutaneous, oral and rectal administration. (A) Subcutaneous IFX injection to DSS‐treated rats. (B) Serum concentration of IFX over time (results are shown as mean ± SD; *n* = 6), (C) total systemic exposure (AUC), and (D) colonic retention after subcutaneous injection. IFX was not detected in the serum of DSS‐treated rats, neither after (E) oral nor (F) rectal administration. Colonic concentration of IFX in healthy and DSS‐treated rats after (G) oral and (H) rectal administration. Concentration of IFX in the colonic stools of healthy and DSS‐treated rats after (I) oral and (J) rectal administration. Concentration of IFX in (K) colonic stools and (L) excreted stools of healthy rats after rectal administration. (M) Ex vivo experimental setup and (N) IFX tissue concentration after six‐hour incubation of oral and rectal formulations. In panels C, D, G, H, I, J, K, L, and N, results are shown as single point values ± SD. Statistical significance was evaluated by one‐way ANOVA with Tukey's post hoc analysis. Adjusted *p‐*values are reported above the comparison.

The systemic absorption of mAbs is associated with adverse effects [42]. To minimize these risks, our formulations were designed for localized mAb delivery, aiming for high drug concentrations in the inflamed colon and limited systemic exposure. Accordingly, systemic IFX levels were below the quantification limit of 0.1 µg/mL at all measured time points following both oral and rectal administrations, indicating limited systemic exposure (Figure 4E,F). Whereas the oral dose was selected based on the systemic reference dose, the rectal dose was guided by our previous study, which showed that half of the LMP gel was excreted after administration [24].

Colonic retention was assessed 12 h post‐rectal administration and 24 h post‐oral administration in both healthy and DSS‐treated rats (Figure 4G,H). After oral administration, colonic IFX levels in healthy rats were slightly higher for both InflixiCaps and PowderCaps, although the differences were not statistically significant (*p* > 0.05). Similarly, rectal administration resulted in higher IFX tissue concentrations in healthy rats compared with DSS‐treated counterparts. Stool analysis revealed lower IFX levels in colitic animals (Figure 4I,J). To assess retention over time, colonic tissue and stool samples were collected from healthy rats 12 and 24 h after rectal administration of InflixiGel and enema. IFX tissue concentrations were comparable at both time points for both formulations (Figure S11). However, higher IFX amounts were detected in the colon contents at 24 h for InflixiGel, suggesting prolonged local retention (Figure 4K). Analysis of excreted stools collected throughout the experimental period further showed higher IFX levels in rats treated with enema (Figure 4L), consistent with its lower mucoadhesion (Figure 3E).

The lower IFX levels detected in colonic tissues of DSS‐treated rats might result from rapid binding and consumption of the antibody by TNF‐α, which is overexpressed in the inflamed intestinal mucosa of IBD patients [43, 44, 45]. To test this hypothesis, we harvested colons from rats and assessed the retention of IFX in an ex vivo setup, where formulations were added inside the colon, and both ends of the tissue were sealed (Figure 4M). In the ex vivo setup, all formulations led to markedly higher tissue concentrations of IFX compared to in vivo (Figure 4N). Non‐LMP formulations (PowderCaps and enema) showed higher IFX tissue retention, highlighting the controlled release profile of LMP‐based gels (InflixiCaps and InflixiGel).

The rapid GI transit of InflixiCaps, reaching the colon within 6 h, together with the low IFX levels detected in colonic tissues and stools, supports a daily dosing regimen for efficacy studies. Similarly, IFX concentrations in inflamed colonic tissues and stools following rectal administration, along with the higher excretion observed with the enema, also support a daily dosing frequency of rectal formulations.

### 2.5 Prophylactic Efficacy of InflixiCaps and InflixiGel in DSS‐Induced Colitis Rats

The protective effects of InflixiCaps and InflixiGel against colonic inflammation were investigated using a DSS‐induced colitis model in Sprague–Dawley rats. Unlike other chemically induced colitis models, DSS models allow modulation of disease severity through concentration and dosing frequency [46]. However, the inflammatory response is modulated by the intestinal microbiome [47]. DSS has been employed at a range of concentrations (4%–8%) to induce acute colitis in Sprague–Dawley rats [35, 48, 49]. In this study, DSS administration was standardized by using 4% and 6% DSS for seven days to induce moderate and severe colitis, respectively. In both inflammation severity models, no significant reduction in body weight was observed across all treatment groups (<5% reduction; Figure S12).

In the moderate colitis model, untreated animals developed soft stools with occasional diarrhea and bleeding, as well as progressively increasing disease activity index (DAI) values, which were similar between untreated males and females (Figure S13). Both InflixiGel and InflixiCaps produced a modest early reduction in DAI, which became statistically significant from day five onward, resulting in significantly lower DAI values compared with untreated animals (Figure 5A). On day seven, fecal lactoferrin, a validated marker of intestinal inflammation [50, 51], was significantly elevated in untreated rats relative to naïve controls (*p* = 0.0353) and significantly reduced by both localized IFX formulations (Figure 5B). Colon length, an indicator of DSS‐induced tissue damage, was reduced in untreated animals, particularly in males (Figure S14). Treatment with InflixiGel and InflixiCaps trended toward preservation of colon length; however, this difference was not statistically significant (*p* > 0.05; Figure 5C). In this model, tissue concentrations of TNF‐α were comparable across all experimental groups (Figure 5D). It should be noted that the inflammatory response to DSS is influenced not only by the DSS concentration and exposure duration, but also by factors such as animal strain, sex, and microbiome composition. In particular, progressive upregulation of TNF‐α with increasing DSS concentrations has been reported [52]. Importantly, TNF‐α is not the sole driver of inflammation in DSS‐induced colitis. Histological changes in the colonic mucosa, including crypt loss and increased inflammatory cell infiltration, are commonly observed alongside increased expression of multiple pro‐inflammatory cytokines [52]. Therefore, comparable tissue concentrations of TNF‐α across groups do not necessarily indicate an absence of differences in overall colonic inflammation.

**FIGURE 5:**
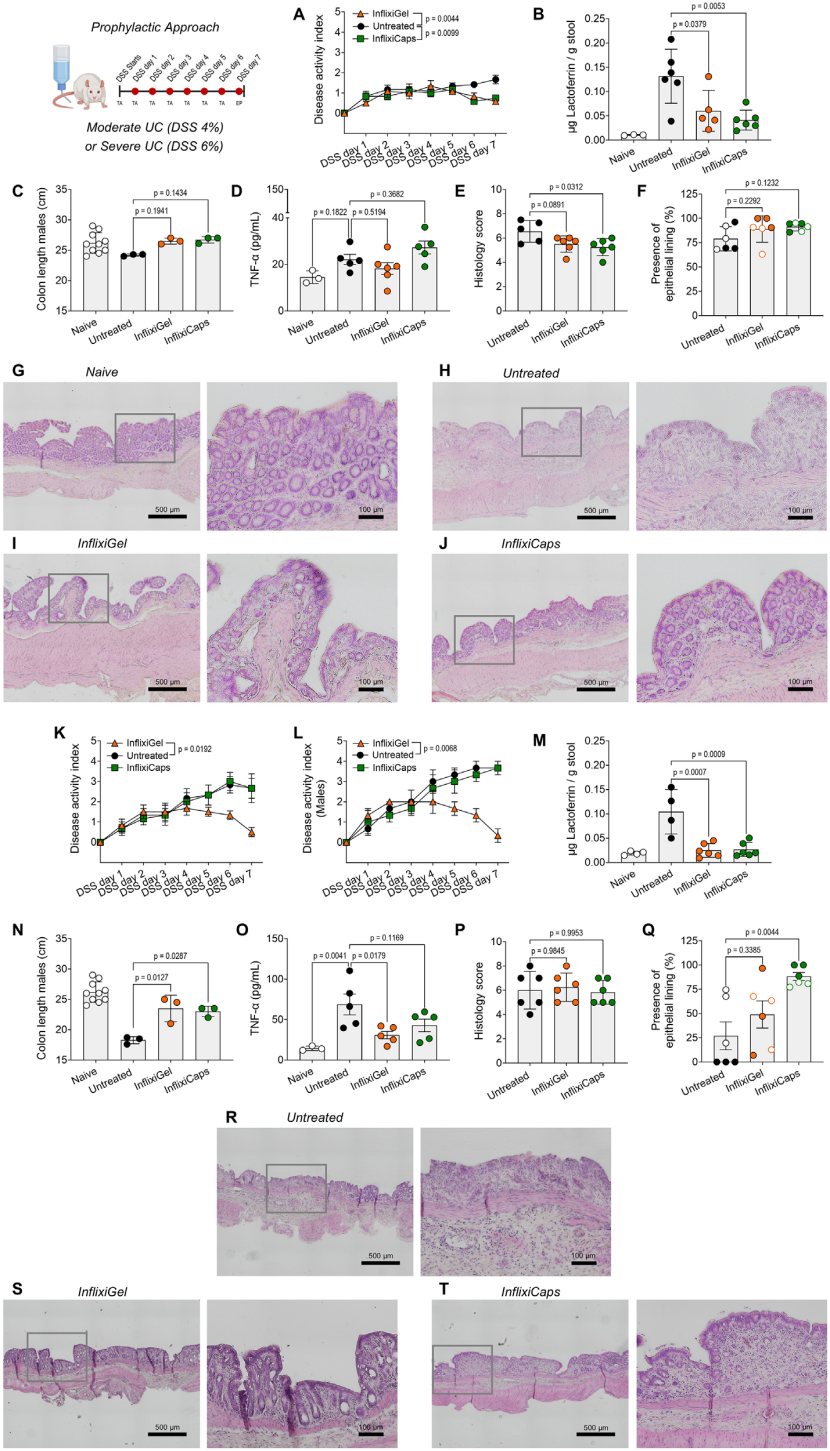
Prophylactic efficacy of InflixiGel and InflixiCaps in rats with moderate and severe DSS‐induced colitis. Animals received 4% DSS in A–J. (A) Daily disease activity index (results are shown as mean ± SD; *n* = 6). (B) Fecal lactoferrin concentration, (C) colon length, and (D) colon TNF‐α concentration in naïve, untreated, and treated rats after DSS. (E) Histology score and (F) presence of epithelial lining in males (closed symbols) and females (open symbols) after DSS. In panels B–F, results are shown as single point values ± SD. Representative histology pictures of (G) naïve, (H) untreated, (I) InflixiGel‐treated, and (J) InflixiCaps‐treated animals after DSS. Animals received 6% DSS from K‐ T. Daily disease activity index of (K) female and male rats (results are shown as mean ± SD; *n* = 6) and (L) male rats (results are shown as mean ± SD; *n* = 3). (M) Fecal lactoferrin concentration, (N) colon length, and (O) colon TNF‐α concentration in naïve, untreated, and treated rats after DSS. (P) Histology score and (Q) presence of epithelial lining in males (closed symbols) and females (open symbols) after DSS. In panels M–Q, results are shown as single point values ± SD. Representative histology pictures of (R) untreated, (S) InflixiGel‐treated, and (T) InflixiCaps‐treated animals after DSS. Sample size was *n* = 6 rats/group. Statistical significance was evaluated using linear mixed‐effects (A,K,L), and one‐way ANOVA with Tukey's post hoc analysis (B–F,M–Q), for which outliers were identified by Grubbs’ test (*α* = 0.05) and removed in B, D, E, M, and O. Adjusted *p*‐values are reported above the comparison. TA: treatment administration, EP: endpoint.

Histologically, InflixiGel and InflixiCaps mitigated epithelial injury, preserved crypt architecture, and maintained goblet cell populations, with reduced lymphocytic infiltration and submucosal edema compared to untreated rats (Figure 5E). In addition, the colonic mucosa was better preserved in the treated groups, although not statistically different than the untreated group (Figure 5F). Compared to naïve animals, untreated DSS‐exposed animals exhibited severe mucosal damage characterized by marked crypt distortion, extensive goblet cell depletion, and fragmented lining epithelium (Figure 5G,H). Light lymphocytic infiltration was observed in both the mucosa and submucosa, together with prominent submucosal edema. InflixiGel‐treated rats showed moderate crypt distortion and detectable goblet cells (Figure 5I). Mild lymphocytic infiltration was mainly confined to the mucosa, whereas submucosal edema remained comparable to the other groups. Similarly, InflixiCaps‐treated animals exhibited preserved crypt architecture and maintained goblet cell populations (Figure 5J). Mild lymphocytic infiltration was primarily restricted to the mucosa, and submucosal edema was observed at levels comparable to those in the other treatment groups. Together, these results demonstrate that both strategies for localized IFX delivery provide significant protection against DSS‐induced colonic inflammation.

In the severe colitis model, untreated animals exhibited markedly higher DAI values than those observed in the moderate colitis model, due to persistent diarrhea and frequent rectal bleeding. In this setting, InflixiCaps did not significantly attenuate DAI, whereas InflixiGel robustly reduced DAI from day five onward (Figure 5K). These results highlight the advantage of rectal administration for achieving high local drug concentrations. Disease severity was sex‐dependent in this model, with males developing more severe disease than females (Figure S15). In males, InflixiGel nearly normalized DAI by day seven (Figure 5L), highlighting the strong efficacy of the rectal formulation under severe inflammatory conditions. These findings align with the localized action of IFX within the inflamed colonic mucosa. Our previous study demonstrated that an MLO‐based blank carrier lacks intrinsic therapeutic activity in a rodent model of DSS‐induced colitis [24]. Nevertheless, a potential mucosal protective effect exerted by the vector itself cannot be entirely excluded as a contributing factor to the overall efficacy of the formulation. Fecal lactoferrin levels were elevated in untreated animals and reduced to near‐naïve levels in InflixiGel and InflixiCaps treatment groups (Figure 5M). Untreated males exposed to 6% DSS displayed significantly shorter colons than naïve animals, whereas this effect was less evident in females (Figure S16). Accordingly, the colon length was analyzed in males (Figure 5N). Both InflixiGel and InflixiCaps preserved colon length relative to untreated controls, with values comparable to those of naïve animals. In contrast to the moderate colitis model, severe colitis was associated with a marked TNF‐α component and, under these conditions, both InflixiGel and InflixiCaps significantly reduced tissue TNF‐α concentrations (Figure 5O), restoring levels to those observed in naïve controls.

Epithelial damage and immune cell infiltration scores were comparable among groups (Figure 5P), demonstrating that improvements in macroscopic outcomes and biomarkers were not accompanied by corresponding histological healing. However, qualitative histological analysis revealed differences in mucosal integrity among treatment groups. InflixiCaps markedly preserved the epithelial lining compared with untreated animals (Figure 5Q). Particularly, untreated males exhibited complete loss of the epithelial lining. Additionally, the untreated group displayed crypt distortion, goblet‐cell depletion, and widespread lymphocytic infiltration (Figure 5R). In contrast, InflixiGel preserved crypt architecture, maintained goblet cells, and restricted lymphocytic infiltration to the mucosa (Figure 5S). InflixiCaps produced a similar protective pattern, with moderate crypt distortion and sporadic goblet‐cell preservation (Figure 5T). Importantly, this histological profile closely resembled that observed in the moderate DSS model, indicating that both formulations effectively protect the colonic mucosa under severe inflammatory stress, with formulation‐specific advantages: InflixiCaps predominantly maintained the epithelial lining, whereas InflixiGel, likely through topical contact with the mucosa, preserved the crypt architecture.

## 3 Discussion

Despite their clinical success in UC treatment, systemically administered mAbs are associated with dose‐limiting toxicity, immunogenicity, and suboptimal drug exposure to the inflamed mucosa. Intravenous or subcutaneous delivery of IFX results in high systemic exposure despite the disease being confined to the colon. Here, we addressed this fundamental mismatch between drug delivery route and disease localization by developing oral and rectal LMP‐based formulations that successfully delivered IFX to the inflamed colon while minimizing systemic absorption. Oral delivery of biologics requires gastro‐resistant protection, as these molecules are rapidly degraded in the stomach [53]. Our research group previously developed a gastro‐resistant 3D‐printed tablet using S80/VitE_LMP [34], which informed the selection of this gel as the protective shell for InflixiPrint. This LMP exhibits mixed lamellar and hexagonal phases, both associated with slower diffusion rates compared to inverse bicontinuous cubic phases [23]. In addition, the S80/VitE_LMP contains only 20% water, limiting the incorporation of hydrophilic molecules within its aqueous channels. In contrast, cubic mesophases allow for higher water content, increased loading of hydrophilic molecules, and faster drug diffusion [23]. However, their narrow water channel diameter can restrict the release of macromolecules [27, 28]. To overcome this limitation, hydration‐modulating additives such as SE have been incorporated into cubic mesophases [26, 27]. Accordingly, the InflixiPrint core was designed using an MLO‐based LMP containing SE at an optimized concentration to enable successful 3D printing while maintaining mechanical stability. Increasing SE content reduced the flow point and improved extrudability. The structural effect of SE on LMPs has been reported [29], and its influence on rheological behavior has been linked to a reduction in apparent viscosity [54], consistent with our findings. Moreover, a direct link between water channel dimensions and structural strength has already been reported, which is in line with our observation [55].

The addition of SE to the lipid mixture has previously been reported to induce significant swelling, accompanied by a phase transition from Pn3m to Im3m [29]. The highly hydrophilic headgroup of SE decreases the critical packing parameter and reduces the curvature of the lipid bilayer toward the aqueous phase, resulting in phase transition and enlarged water channels. However, the curvature of the lipid bilayer is determined not only by the molecular structure of its constituent lipids and additives, but also by external factors such as salts present in the aqueous phase [31, 56, 57]. Here, the addition of 30% SE induced only minor swelling when hydrated with PBS, and a complete phase transition to Im3m was not observed. Negrini et al. [29] hydrated the mesophase using HEPES buffer, which has a weaker charge density compared with PBS. Chaotropic ions, characterized by weaker hydration and lower charge density, have been reported to increase mesophase hydration and the lattice parameter. In contrast, kosmotropic salts, such as Na_2_HPO_4_ in PBS, decrease the lattice parameter by strengthening ion–water interactions, leading to an increased bilayer curvature [58]. These differences explain the overall reduced swelling capacity of our system. Hydration of the mesophase with a 30 mg/mL IFX solution further increased the water channel diameter, and SAXS data revealed a coexistence of Pn3m and Im3m phases under these conditions. We hypothesized that the accommodation of large antibody molecules within the water domains caused frustration in bilayer packing, triggering the transition from Pn3m to Im3m. Conversely, hydration of the mesophase with a highly concentrated IFX solution (100 mg/mL) reduced the water channel diameter. This concentrated solution contained not only more IFX but also a higher level of phosphate salts from the lyophilized powder (NaH_2_PO_4_ and Na_2_HPO_4_), which promoted water channel shrinkage [58].

Lipid‐based solid oral dosage forms have demonstrated clinical and commercial success [59, 60]. However, their formulation remains challenging due to the high viscosity and heat sensitivity of many lipids, which complicate conventional tableting processes [34, 61]. Consequently, most existing lipid‐based solid oral formulations rely on encapsulation, often using gelatine capsules [60]. SSE 3D printing provides an alternative approach for producing lipid‐rich solid oral dosage forms. The printable S80/VitE_LMP developed by our group has 80% lipid content. This formulation was shown to be gastro‐resistant and to undergo digestion in simulated intestinal fluids, forming colloidal structures that enhance the solubility of poorly water‐soluble drugs [34]. In this study, InflixiPrint also maintained its integrity in acidic conditions, confirming gastro‐resistance. Disintegration and subsequent dissolution are critical determinants of oral drug absorption and can represent a rate‐limiting step in the absorption process [62]. While lipid‐based solid oral dosage forms typically include disintegrants and/or surfactants to enable these processes, InflixiPrint was deliberately designed for slow disintegration in intestinal fluids to achieve delayed, colonic drug release. Slower disintegration and dissolution were observed in FaSSIF, which is consistent with its lower bile salt concentration compared to FeSSIF. Therefore, administration under fasted conditions is expected to prolong disintegration and favor a delayed release. Considering that the average small intestinal transit time is approximately 3–4 h [63], the <30% drug release within 6 h suggests that a substantial fraction of IFX remains in the core until it reaches the colon, where complete release can occur. Compared with InflixiPrint, InflixiCaps exhibited higher IFX release in FaSSCoF. This difference likely arises from the pH‐responsive coating on InflixiCaps, which dissolves above pH 7, facilitating a faster release. In contrast, InflixiPrint demonstrated more gradual shell disintegration, resulting in a slower release profile. Scaling down the size of formulations for preclinical studies resulted in only minor changes in their behavior. The slightly faster release observed in FaSSIF for the rat‐sized printlet, compared to the human‐sized one, may be attributed to its thinner shell (0.2 mm vs. 1 mm; Figure S6), which is more rapidly digested in simulated intestinal fluids, resulting in earlier exposure of the IFX‐loaded core. The faster release from PowderCaps compared to InflixiCaps, despite the same coating, is likely due to the absence of the LMP matrix, as IFX is readily available once the PowderCaps coating dissolves, whereas, in InflixiCaps, IFX diffusion is slowed by its encapsulation within the LMP.

The rectal formulation, InflixiGel, demonstrated a high yet gradual IFX release (up to 8 h) in FaSSCoF enriched with lipase, an enzyme present in the inflamed colonic mucosa [37, 38]. These findings indicate that IFX release is enhanced under inflammatory conditions, potentially increasing mAb concentration in inflamed sites. The limited IFX release from the LMP lacking SE (MLO_100_SE_0_), contrary to what was observed for InflixiGel, likely results from lipase‐mediated hydrolysis of the sugar ester [64, 65, 66] and confirms the critical role of SE in enabling inflammation‐triggered release from InflixiGel in the colon. Interestingly, the lipase‐mediated release was less pronounced in PBS, suggesting a synergistic effect of lipase and FaSSCoF in promoting drug release. This highlights the importance of evaluating release kinetics under physiologically relevant conditions to accurately predict the in vivo performance. Beyond the controlled release properties, the SE‐doped LMP enabled mAb encapsulation at high concentrations (30 mg/mL) and effectively prevented protein aggregation and degradation during storage, as evidenced by comparable amounts of active IFX released from the gel after storage. We hypothesized that the 3D mesophase structure is partially responsible for preserving the mAb monomeric form and, therefore, its biological activity. Indeed, LMPs have been widely employed as membrane‐mimetic host matrix for the stabilization, solubilization, and crystallization of membrane proteins [67, 68, 69]. The incorporation of proteins into LMPs has been shown to preserve their enzymatic activity and native conformation [70, 71, 72]. More recently, the development of ultra swollen mesophases has further expanded the application of LMPs by enabling the accommodation and stabilization of proteins with large extracellular domains in a closed conformation [33]. These findings support our hypothesis that IFX can be accommodated within the aqueous channel network of the LMP while retaining its structural and functional integrity. Notably, additional studies are required to assess the long‐term stability of these formulations. We recently validated a dual‐syringe method for LMP production, enabling the aqueous and lipid phases to be mixed within minutes and allowing LMP preparation by the patient or healthcare professional immediately before administration [73]. In addition, 3D printing technologies enable flexible and personalized manufacturing and may support on‐demand production within pharmacy or hospital settings. The combination of these approaches could address potential storage‐related limitations while providing a framework for scalable and decentralized manufacturing of personalized LMP‐based oral and topical formulations.

The oral and rectal delivery of biotherapeutics have been widely investigated; however, no commercial dosage form currently enables localized mAb delivery to the inflamed colon. In particular, conventional rectal formulations show poor retention and insufficient drug absorption. Previously, MLO‐based LMPs demonstrated superior colonic retention in mice compared to enemas [24]. This enhanced retention was attributed to the self‐assembly behavior of the mesophase, as the low‐viscosity lamellar precursor undergoes a transition to a highly viscous cubic mesophase at body temperature, thereby improving formulation retention at the administration site. It has been shown that cubic phases exhibit mucoadhesive properties when formed on wet mucosa, potentially due to dehydration of the mucosa as the precursor phase takes up water during its conversion to the cubic phase [74]. Our findings further demonstrate that incorporation of SE enhances the mucoadhesive properties of the LMP. This effect is likely mediated by weak secondary interactions between the hydrophobic backbone of sugar esters and mucin strands [75], promoting prolonged mucosal adhesion. However, the high viscosity and stiffness of LMPs can limit their use as a standalone oral delivery system [76, 77]. Although we successfully 3D‐printed solid oral dosage forms composed exclusively of LMPs, their administration to small animals remains challenging and restricts in vivo testing. Other studies have encountered similar challenges when delivering LMPs orally and have addressed this by administering a mesophase precursor instead [77, 78]. A previous study showed that cubic mesophases formed in situ after oral administration of lipids containing a poorly water‐soluble drug, resulting in sustained drug release [78]. Another strategy, the same adopted by us, is using enteric capsules filled with LMPs [79]. However, gastric emptying is governed by a complex interplay between physiological and formulation‐related factors and often represents a rate‐limiting step in the absorption of orally administered drugs. Inconsistencies in the gastric emptying of size nine capsules with comparable average weights have been documented in anesthetized rats [80]. To minimize anesthesia‐induced effects on GI motility, our study incorporated a 4 h interval between measurements, as motility impairment has been reported for up to 2 h after isoflurane exposure [81]. Nevertheless, distinct gastric emptying profiles were observed for InflixiCaps and PowderCaps. A previous study reported the hydration and swelling of Eudragit FS30D films in gastric fluids [82]. This behavior potentially allowed water to diffuse into the capsules, thereby hydrating their cores while still preventing drug release. Consequently, mannitol in PowderCaps and LMP in InflixiCaps were hydrated. Owing to its greater swelling capacity, LMP absorbs more water than powder alone (mannitol), resulting in increased weight. We further characterized this behavior in vitro by incubating InflixiCaps and PowderCaps in water. Whereas PowderCaps remained floating at the surface, InflixiCaps sank, providing a possible mechanistic explanation for the faster gastric emptying observed for InflixiCaps (Figure S17). InflixiCaps remained intact throughout the upper gastrointestinal tract and reached the caecum within 6 h. This observation is consistent with our in vitro data, which demonstrated the gastro‐resistant properties of the formulation, with no detectable drug release for up to 5 h in simulated gastrointestinal media. The gastro‐resistant behavior can be attributed to the Eudragit FS30D coating used for the capsules, consistent with similar approaches reported in previous studies [83, 84]. Eudragit FS30D is an anionic copolymer of methacrylic acid, methyl acrylate, and methyl methacrylate that dissolves above pH 7, thereby preventing capsule disintegration in the stomach and small intestine and enabling coating dissolution and subsequent drug release in the ileum and colon [85].

Infliximab can be administered by intravenous infusion or subcutaneous injection. Clinical studies have shown that subcutaneous IFX provides a more stable pharmacokinetic profile than intravenous IFX, characterized by a more sustained systemic exposure and higher drug concentrations [86]. In addition, subcutaneous IFX has demonstrated clinical remission rates comparable to those achieved with intravenous IFX [87]. Therefore, we used the commercially available subcutaneous IFX formulation (Remicade) as a systemic reference for comparison with our localized delivery strategies. The pharmacokinetic profile observed following subcutaneous injection was consistent with previously reported data for anti‐TNF antibodies in rodents [88, 89] and humans [90]. Elevated colonic levels of IFX in inflamed tissues have been attributed to TNF‐α overproduction and the consequent recruitment of anti‐TNF agents, allowing inflamed tissues to act as drug reservoirs [91]. However, under severe inflammatory conditions, reduced tissue levels of anti‐TNF agents have been reported, likely due to insufficient anti‐TNF to neutralize TNF‐α [92]. We hypothesized that the lower IFX concentrations detected in colonic tissues and stools of DSS‐treated rats following oral and rectal administrations result from its immediate consumption by TNF‐α in the colonic mucosa. This hypothesis was supported by ex vivo experiments using inflamed colonic tissues, where higher IFX retention was observed. TNF‐α levels in the tissue rapidly decrease after harvesting, explaining the increased colonic IFX concentrations observed ex vivo. These findings demonstrate that IFX remains structurally stable and effectively penetrates inflamed colonic tissues. Additionally, the controlled‐release properties of the LMP‐based systems were evidenced by the lower IFX colonic retention from InflixiCaps and InflixiGel compared with the non‐LMP formulations. However, the experiment was conducted in a closed system with both ends of the colon sealed, which enhances formulation‐tissue contact and promotes maximal retention. Particularly, the high IFX concentrations in the enema‐treated colon are not representative of in vivo conditions, where this formulation leaks rapidly after administration and shows poor colonic retention.

Patients who responded to anti‐TNF agents for induction of remission are recommended to continue treatment with the same drug for maintenance of remission [8]. In clinical practice, IFX is often the biologic of choice; however, maintenance therapy requires repeated intravenous infusions every eight weeks [93], placing a substantial burden on both patients and healthcare systems. Self‐administered formulations are strongly preferred by patients during maintenance therapy, as they enhance patient autonomy, facilitate integration into daily routines, and reduce the burden of frequent clinical visits [94]. While subcutaneous administration offers a more convenient, at‐home option compared with intravenous infusion, it is associated with a similar immunogenicity profile [95], which can lead to impaired therapeutic response and adverse effects [96]. It has been reported that IFX concentrations during the maintenance phase exert the strongest influence on immunogenicity [97]. Immunogenicity can manifest through the generation of anti‐drug antibodies, which may reduce or even neutralize the therapeutic effects of mAbs [98]. Additionally, anti‐drug antibodies have been associated with an increased incidence of treatment‐related adverse events, including infusion and injection‐site reactions [98, 99]. In this context, localized therapeutic approaches, as proposed here, could potentially mitigate immunogenicity and adverse effects associated with systemic treatments by enabling high drug concentrations at inflamed sites while reducing systemic exposure.

The protective effects of InflixiCaps and InflixiGel against colonic inflammation were evaluated using a prophylactic treatment protocol. In this approach, treatments are administered before the development of acute inflammation. Although clinical therapy is typically initiated during an active flare to induce remission, it is routinely continued long‐term to maintain remission and prevent disease relapse. The prophylactic protocol was therefore intended to reflect this maintenance setting by assessing the efficacy of the formulations to prevent or attenuate inflammation when treatment is initiated before active inflammation becomes established. Furthermore, replicating the clinical treatment timeline in preclinical models presents significant challenges, as human therapies often extend over months, a duration that is logistically difficult to reproduce in DSS‐induced colitis models in rodents. The prophylactic treatment protocol also reduces the potential stress and discomfort associated with administering rectal formulations to rodents after severe colitis has developed, while allowing the biological activity of the formulations to be evaluated in a clinically relevant setting. Nevertheless, this approach has inherent limitations, as it evaluates the prevention or attenuation of tissue damage rather than the resolution of established inflammation. Further studies using treatment initiation after disease onset are required to fully assess the preclinical efficacy of these formulations.

The LMP‐based formulations were evaluated in DSS‐induced models of moderate and severe colitis in Sprague–Dawley rats. Regardless of inflammation severity, body weight could not be used as a reliable readout of disease activity, as it remained stable or even increased across all groups throughout the DSS cycle. This observation can be explained by the growth curve of Sprague–Dawley rats, which are still growing at eight weeks of age; therefore, disease manifestation may be reflected by stagnation or a slower weight gain rate instead of weight loss. Similar findings have been previously reported [100, 101]. In the moderate inflammation model, both formulations consistently improved all disease‐related endpoints. Histological analysis revealed preservation of luminal epithelial lining, retention of goblet cells, and reduced crypt disruption in the LMP‐treated groups. Additionally, mild inflammatory cell infiltration could explain the minor increase in tissue concentrations of TNF‐α in the untreated group. In the severe inflammation model, the therapeutic benefits of LMP‐based formulations became even more evident at the macroscopic and biochemical levels, with prevention of colon shortening and marked reductions in DAI, fecal lactoferrin levels and tissue TNF‐α concentrations. TNF‐α is a central mediator of intestinal inflammation and one of the most widely used molecular markers in the DSS model of colitis. DSS‐induced epithelial injury leads to activation of innate immune pathways and subsequent production of pro‐inflammatory cytokines, among which TNF‐α plays a key role in amplifying mucosal damage, leukocyte recruitment, and barrier dysfunction [102, 103]. Numerous studies have demonstrated that TNF‐α expression correlates with disease severity in DSS colitis models [52, 104]. Consistent with these reports, in our moderate colitis model, tissue concentrations of TNF‐α remained comparable across all groups, indicating that inflammation at this stage is largely driven by epithelial injury and innate immune activation, rather than sustained TNF‐α overproduction. In contrast, in the severe colitis model, TNF‐α was a dominant inflammatory mediator, and its tissue levels were markedly reduced toward those observed in naïve animals with both InflixiCaps and InflixiGel treatments. This severity‐dependent modulation of TNF‐α aligns closely with previously described DSS models and supports the biological relevance of our system, demonstrating that therapeutic TNF‐α neutralization becomes particularly effective under conditions of advanced mucosal damage and heightened inflammatory burden.

Despite macroscopic improvements in the severe colitis model, conventional histological scoring, primarily based on epithelial damage and immune cell infiltration, did not reveal significant differences between untreated and treated groups. The apparent mismatch between macroscopic improvement and histological scores under severe inflammation has been previously reported in DSS‐induced colitis models [105, 106, 107] and likely reflects the inability of short‐term anti‐inflammatory treatments to fully reverse epithelial damage once severe injury has occurred. Chronic mucosal injury in UC has been reported to interrupt or even destroy the normal regeneration and repair processes of damaged intestinal epithelial cells [108, 109], and the 6% DSS model induces particularly aggressive mucosal injury. Under these conditions, even the commercially available subcutaneous injection used as a positive control was unable to reduce DAI, prevent colon shortening, or restore colonic epithelial integrity (Figures S18,S19). Higher concentrations of anti‐TNF agents have been detected in the inflamed mucosa of IBD patients, suggesting increased local TNF‐α production and consequent recruitment of anti‐TNF agents to inflamed tissues [91, 92, 110]. However, the presence of anti‐drug antibodies, which can develop during systemic treatment, has been associated with lower tissue concentrations of IFX [111]. Yoshihara et al. [110] demonstrated that, among patients with high serum trough concentrations of anti‐TNF agents, those with higher tissue concentrations tended to have more favorable long‐term outcomes than those with lower tissue concentrations. Similarly, Yarur et al. [92] suggested that persistent inflammation in some patients may be associated with a high tissue TNF‐to‐anti‐TNF ratio despite therapeutic serum anti‐TNF concentrations. Together, these findings highlight the importance of achieving sufficient tissue exposure to anti‐TNF agents.

Given the localized nature of UC, we hypothesized that localized anti‐TNF delivery could represent a potential therapeutic strategy by achieving high drug concentrations directly at inflamed sites while reducing systemic exposure and potentially mitigating immunogenicity. Notably, both InflixiGel and InflixiCaps outperformed the systemic positive control, highlighting the advantage of localized IFX delivery in preserving mucosal barrier function under conditions of severe inflammation. Importantly, the qualitative histological features preserved by InflixiGel and InflixiCaps (presence of continuous epithelial lining, retention of goblet cells, and reduction in crypt destruction) are mechanistically consistent with the known mucosal actions of IFX. In a seminal ex vivo and in vitro study, IFX was shown to directly promote mucosal healing [112]. The preservation of goblet cells and the luminal epithelial layer observed in our LMP‐treated animals, therefore, represents a biologically meaningful therapeutic outcome, as these structures are essential for preventing bleeding, bacterial translocation, and further inflammatory amplification in UC [108]. In this context, the ability of InflixiGel and InflixiCaps to preserve goblet cells and epithelial continuity likely explains the marked reductions in fecal lactoferrin and disease activity observed in both moderate and severe models. These barrier‐preserving effects suggest that LMP‐based colonic delivery not only suppresses inflammation but actively promotes mucosal healing, which is the strongest predictor of sustained remission and reduced hospitalization in UC patients. Previous studies have demonstrated that agents that promote hydration and restore colonic mucosal barrier function can improve UC outcomes by enhancing epithelial integrity and protecting the mucin layer [113]. These observations underscore the importance of maintaining mucosal structure in mitigating inflammation and suggest that localized delivery strategies that preserve or protect the epithelial lining, such as our LMP‐based formulations, may offer therapeutic advantages.

## 4 Conclusion

In this study, we demonstrated the previously underexplored potential of LMPs as versatile and effective platforms for targeted mAb delivery to the inflamed colon. By doping MLO‐based LMPs with SE, we successfully enlarged the aqueous channel diameter to enable efficient IFX release while simultaneously modulating gel rheology. This dual functionality proved critical for enabling SSE 3D printing of the oral dosage form InflixiPrint and facilitating the practical administration of the rectal formulation InflixiGel. LMP‐based formulations successfully reached the colon following oral administration of the enteric capsules InflixiCaps and exhibited strong mucoadhesion after rectal administration of InflixiGel. These properties translated into prophylactic effects in a DSS‐induced colitis model, with significant attenuation of disease activity, inflammatory biomarkers, and histological damage. Overall, this study provides a new biocompatible and adaptable LMP‐based delivery platform for colon‐targeted drug delivery. By minimizing systemic exposure while preserving local drug activity, this approach represents a promising strategy for localized biotherapeutic delivery in UC and provides a basis for further translational investigation.

## 5 Materials and Methods

### 5.1 Study Design

This study was designed to explore the potential of LMP‐based drug delivery systems for the treatment of UC. We investigated the incorporation of a swelling agent into MLO‐based mesophases and the resulting modulation of their rheological properties and drug delivery features. The developed formulations were designed to rapidly adhere to the inflamed intestinal mucosa and enable controlled mAb release. The LMP‐based formulations were first characterized in vitro. Subsequently, the efficacy of the localized drug delivery systems was evaluated in a DSS‐induced colitis model in rats in comparison with subcutaneous IFX, as a systemic reference.

### 5.2 Preparation of LMPs

The LMP composed of S80 and vitamin E (VitE) was prepared as previously described [34], and the detailed methodology is provided in the Supporting Information. The LMP composed of MLO and SE was prepared by melting and mixing the lipid with different percentages of SE (Table S2) in a magnetic stirrer for 1 h (500 rpm, 40°C). The MLO/SE lipid mixture (50% w/w) was hydrated with PBS or IFX solution (50% w/w) using the dual‐syringe method as previously described [73], with detailed methodology provided in the Supporting Information. MLO_100_SE_0_ was produced by mixing MLO (60% w/w) and PBS or IFX solution (40% w/w). The preparation of IFX solutions and their quantification by HPLC and ELISA are described in the Supporting Information.

### 5.3 Semisolid Extrusion 3D Printing

The design of the 3D‐printed tablets was created in AutoCAD 2024.1.2 (Autodesk, USA). The shell and core of the design were exported as.stl files, which were arranged in Slic3r, and the model was sliced to be printed with two LMPs. A Bio‐X bioprinter (Cellink, Sweden) with a pneumatic thermoplastic printhead was used for 3D printing the tablets. To ensure optimal results, approximately 3 g of each formulation was placed into a pneumatic syringe and centrifuged at 4430 × *g* for 15 min to remove air bubbles.

The optimal printing parameters were determined using a full factorial DoE generated by Minitab 18.1 software, with detailed methodology provided in the Supporting Information. The printing parameters for the S80/VitE_LMP were pressure of 65 kPa, print speed of 3 mm/s, extruder temperature of 37°C, and nozzle diameter of 27G. The printing parameters for the MLO/SE_LMP were a pressure of 110 kPa, a print speed of 3 mm/s, an extruder temperature of 35°C, and a nozzle diameter of 22G. For printing the core of the rat dosage form, a pressure of 160 kPa and a nozzle diameter of 20G were used. The tablets were stored at 2°C–8°C until use. The core was also printed separately to determine the core weight and, therefore, the correct dose. The weight of the full printlet was also determined.

### 5.4 Rheology

A stress‐controlled rheometer (Modular Compact Rheometer MCR 72, Anton Paar, Graz, Austria) was used in cone‐plate geometry (0.993° angle and 49.942 mm diameter) to investigate the rheological properties of the LMPs. Viscosity‐shear rate curves were measured at 25°C, and amplitude strain sweep measurements were performed at a frequency of 10 Hz and strain percentages between 0.01% and 100% to determine both the LVR and the flow point.

### 5.5 Small Angle X‐Ray Scattering (SAXS)

SAXS measurements were performed on a Bruker AXS Micro using a microfocused X‐ray source (λ Cu Kα = 1.5418 Å) operating at a voltage of 50 kV and a filament current of 1000 µA. The diffracted X‐ray signals were collected using a 2D Pilatus 100 K detector. The scattering vector was calibrated using silver behenate. Data were collected and azimuthally averaged using the Saxsgui software to yield 1D intensity versus scattering vector Q, with a Q range from 0.001 to 0.5 Å^−1^. For all measurements, the samples were placed inside a stainless‐steel cell between two thin, replaceable mica sheets and sealed with an O‐ring, with a sample volume of 10 µL and a thickness of ∼1 mm. Measurements were performed at 37°C, and the scattered intensity was collected over 30 min.

### 5.6 Production of Capsules

Gelatine capsules (size 4 for human dosing and size 9 for rat dosing) were filled with MLO_70_SE_30_ LMP and coated by dipping the capsules into Eudragit FS30D for 10 s and drying at room temperature for 10 min (five cycles). Capsules were filled with mannitol and IFX solution (10 µL, 80 mg/mL) and coated with Eudragit FS30D following the same procedure described above. The volume of the IFX solution was adjusted according to the weight of the rats to achieve an IFX dose of 2 mg/kg body weight.

### 5.7 Disintegration Studies of the Oral Dosage Forms

Disintegration was assessed using an automated system (Agilent G7962A) in 900 mL of medium at 37°C. InflixiPrints (*n* = 9) were incubated in HCl 0.1 m for 1 h and then transferred to PBS, FaSSIF, or FeSSIF (*n* = 3). Disintegration was visually assessed at 15, 45, 75, and 105 min.

### 5.8 Dissolution Studies of the Oral Dosage Forms

Dissolution experiments were performed using a basket apparatus (ERWEKA DT 126 light, Apparatus I). InflixiPrints were tested individually in 800 mL of medium at 37°C and 150 rpm, with incubation in HCl 0.1 m for 2 h followed by 6 h in PBS, FaSSIF, or FeSSIF (*n* = 3). Samples were collected at the end of each phase, concentrated using 50 000 MWCO Amicon Ultra centrifugal filters, and IFX concentrations were determined by HPLC and ELISA. Using the same experimental setup, the dissolution profile of InflixiPrint was compared with that of coated capsules in HCl (2 h), FaSSIF (6 h), and FaSSCoF (1 h) (*n* = 3).

### 5.9 Lipolysis Assay of the 3D‐Printed Oral Dosage Form

In vitro lipolysis assays were performed following previously described methods with minor modifications [114], and the detailed methodology is provided in the Supporting Information.

### 5.10 Release Experiments of the Rectal Dosage Form

InflixiGel (100 mg, 1.5 mg IFX) was weighed inside metallic baskets, placed inside Falcon tubes containing 5 mL of release media (PBS with or without lipase (5000 U/mL), FaSSCoF with or without lipase (5000 U/mL) or FeSSCoF) and incubated at 37°C and 100 rpm for 8 h (*n* = 3). The release medium was collected at 1, 2, 4, and 6 h and replaced with fresh 5 mL of buffer. The aliquots were concentrated in a 50 000 MWCO Amicon Ultra centrifugal filter, and IFX concentration was determined by ELISA.

### 5.11 Ex Vivo Experiments

Oral formulations (InflixiCaps or PowderCaps, one capsule inside each colon sample) and rectal formulations (200 mg of InflixiGel or 200 µL of enema inside each colon sample) were added inside colon samples (5 cm) from DSS‐treated rats, 0.5 mL of FaSSCoF enriched with lipase was added, and both extremities were closed with surgical stitches (*n* = 3 per treatment). Colon samples were incubated in FaSSCoF enriched with lipase (5000 U/mL) for 6 h at 37°C and 100 rpm. The colons were opened longitudinally, rinsed with 1 mL PBS, and homogenized with 2 mL PBS using a Polytron PT 2500 E homogenizer (Kinematica, Switzerland). Homogenates were centrifuged at 5000 × *g* for 10 min at 4°C, and IFX concentration in the supernatants was quantified by ELISA according to the manufacturer's instructions (HUMB00005, AssayGenie).

### 5.12 In Vivo Experiments

All animal experiments were performed in strict accordance with the national and institutional guidelines for the care and use of laboratory animals. The experimental protocol was reviewed and formally approved by the Cantonal Veterinary Office of Bern (Switzerland). Approval number BE89/2024. The study complied with the PREPARE and ARRIVE guidelines. Detailed animal housing conditions are provided in the Supporting Information. Animal experiments were conducted using eight‐week‐old Sprague–Dawley rats (Janvier Laboratories, France). Rats were randomly allocated to different treatment groups on the experimental day. Each group included equal numbers of male and female animals, with sex included as a blocking factor in the statistical analysis. Because disease severity was sex‐dependent, with males developing more severe disease, sexes were analyzed separately in Figure 5 (panels C, L and N).

### 5.13 Progression of InflixiCaps and PowderCaps Through the GI Tract After Oral Gavage

GI transit following oral gavage was evaluated in healthy rats for InflixiCaps and PowderCaps loaded with barium sulfate (3 mg/capsule), incorporated either into the LMP or mannitol matrix, respectively. Capsule progression along the GI tract was assessed immediately after administration (*t* = 0) and at 4 and 6 h post‐gavage. In preliminary studies, rats were administered capsules without an additional liquid contrast agent. The capsules were clearly observed, but it was difficult to determine their location, as the GI tract was not highlighted. Therefore, 1 mL of a liquid contrast agent (Ultravist 300, Schering AG, Germany; 1 mL contains 0.623 g iopromide, 300 mg I/mL) was given orally to rats by gavage under isoflurane anesthesia at different time points and administered together with the oral dosage forms. Oral gavage was performed using a rat capsule dosing tube (FTPU‐C9‐85, Instech).

Imaging: CT imaging was carried out using the SmART+ (Small Animal Radiotherapy System), Precision X‐Ray, Inc. (Madison, CT, USA) equipped with a 0.4 mm focal spot X‐ray source and calibrated to a source‐to‐isocenter distance of 30.7 cm and a source‐to‐detector distance of 64.5 cm. Animals were anesthetized with isoflurane (5% in 100% O_2_ at 1 L/min) delivered via a nose cone and positioned in a stereotactic frame. For each scan, the SmART+ System performed a 360° rotation around the stationary animal, with the X‐ray source and detector rotating together to acquire projections from multiple angles. These projections were reconstructed into a 3D cone‐beam CT dataset, providing high‐resolution anatomical information for accurate localization of the capsule within the GI tract.

### 5.14 Colonic Adhesion of InflixiGel After Rectal Administration

Healthy animals were fed an alfalfa‐free diet 72 h before experiments to suppress autofluorescence in the abdominal region and reduce background signals. Immediately before acquiring the first image, the anogenital fur was shaved off to allow accurate dosing and imaging. InflixiGel and enema were loaded with DiD dye (0.02 mg/100 mg of formulation), and 150 mg of the formulation (*n* = 6) was administered rectally to healthy animals under inhalation anesthesia (5% isoflurane in 100% O_2_ at 1 L/min). Rectal administration was performed using sterile, disposable, flexible PTFE cannulas (Sigma CAD‐9920) attached to a syringe containing the formulation. Animals were euthanized with a pentobarbital injection (150 mg/kg intraperitoneal) at 8 h (*n* = 3) and 24 h (*n* = 3) post‐rectal administration. Post‐mortem, the colons were harvested and freshly imaged. Backgrounds (untreated tissue samples) were measured at each time point (*n* = 3). The intensity of the fluorescent signal was measured using an IVIS SpectrumCT In Vivo Imaging System (PerkinElmer, MA, US) equipped with a 650 nm emission filter. The focus was kept stable using a subject height of 1.5 cm, and the temperature of the chamber was set at 37°C. Analysis of images (set with a fixed counts scale from 1000 to 40 000 and acquired with a 0.2 s exposure time) was computed by first defining the region of interest (ROI), which was kept consistent across images, and then summing all counts for all pixels inside the ROI (Total Fluorescence Counts‐ TFC; photons/s). Colonic adhesion profiles were obtained by plotting the TFC as a function of time (h).

### 5.15 Blood and Tissue Pharmacokinetics

Blood: IFX concentration in the blood after administration of the formulations was evaluated in DSS‐treated rats by giving free access to drinking water supplemented with 5.5% (w/v) DSS for 5 days, after which plain water was provided. Animals were randomly allocated to different treatment groups (*n* = 6 rats/group), and on the fifth day of DSS, each animal received one of the following treatments: InflixiCaps, PowderCaps, InflixiGel, enema, subcutaneous injection. The dose for rectal formulations was 5 mg/kg, and for subcutaneous injection and oral formulations was 2 mg/kg. Blood (110 µL) was collected in BD Microtainer serum tubes from the lateral tail vein at 0.5 h, 4 h, 8 h, 24 h, 2 days, 4 days, 7 days, 15 days, and 22 days after treatment administration and allowed to clot by leaving it undisturbed at room temperature for 15 min. Samples were centrifuged at 4°C and 1500 × *g* for 10 min. The serum was collected and stored in cryotubes at −20°C until drug quantification by ELISA according to the manufacturer's instructions (HUMB00005, AssayGenie). AUC 0–22 days was calculated using a noncompartmental analysis with intravascular administration as a model. After the last sampling point, animals were euthanized with CO_2,_ and the colon was collected for tissue IFX quantification.

Tissue: Colonic retention of IFX following oral or rectal administration was evaluated in healthy and DSS‐treated rats. Colitis was induced by giving free access to drinking water supplemented with 5.5% (w/v) DSS for five days. On the fifth day of DSS, colitic rats were randomly assigned to one of the following treatment groups: InflixiCaps, PowderCaps, InflixiGel, enema, or subcutaneous IFX injection. Animals were euthanized at predefined time points using pentobarbital (150 mg/kg, intraperitoneal), and colonic tissues and luminal contents were collected for IFX quantification. Rats receiving oral formulations (InflixiCaps or PowderCaps) were euthanized 24 h after gavage (*n* = 4 rats/group). Rats receiving rectal formulations (InflixiGel or enema) were euthanized at 12 and 24 h after administration (*n* = 4/group). Animals receiving subcutaneous IFX were euthanized at 7 and 14 days post‐injection (*n* = 4/group). Healthy rats were randomly assigned to receive InflixiCaps, PowderCaps, InflixiGel, or an enema. Animals receiving oral formulations were euthanized at 24 and 48 h after gavage (*n* = 3/group), whereas animals receiving rectal formulations were euthanized at 12 and 24 h post‐administration (*n* = 3/group).

Following euthanasia, stool samples were collected, suspended in PBS containing protease inhibitors (cOmplete, Roche Diagnostics, Germany), vortexed, and centrifuged at 5000 × *g* for 10 min at 4°C. Supernatants were stored at −20°C until analysis. Colons were rinsed with PBS, cut into small fragments, and homogenized in PBS containing protease inhibitors using a Polytron PT 2500 E homogenizer (Kinematica, Switzerland). Homogenates were centrifuged at 5000 × *g* for 10 min at 4°C, and the supernatants were concentrated using 50 000 MWCO Amicon Ultra centrifugal filters before storage at −20°C. IFX concentrations in stool and tissue extracts were quantified by ELISA according to the manufacturer's instructions (HUMB00005, AssayGenie).

### 5.16 Therapeutic Efficacy Evaluation Following a Prophylactic Approach

Prophylactic therapeutic efficacy of oral and rectal formulations was evaluated in a DSS‐induced colitis model in rats. Moderate and severe colitis were induced by providing free access to drinking water supplemented with 4% and 6% (w/v) DSS for seven days, respectively. Animals were randomly allocated to different treatment groups (*n* = 6 rats/group), including subcutaneous IFX as a systemic reference, the oral formulations InflixiCaps and PowderCaps, the rectal formulations InflixiGel and enema, and an untreated control group. Empty MLO‐based LMPs have previously been shown to have no intrinsic mucosal‐healing activity [24]. Therefore, an empty gel control was not included in the present study to avoid duplicating existing evidence and requiring additional animal use, in accordance with the Swiss Animal Welfare Act (TSchG), the Swiss Animal Welfare Ordinance (TSchV), and the legally embedded 3R principles (replacement, reduction, and refinement).

On the first day of DSS supplementation, each animal received one of the following treatments: subcutaneous injection (single administration, 2 mg IFX/kg), InflixiCaps or PowderCaps (daily administration for seven consecutive days, 2 mg IFX/kg), InflixiGel or enema (daily administration for seven consecutive days, 5 mg IFX/kg). Following each administration, animals were monitored for 1 h. In the rectally treated groups, if formulation excretion was observed within this period, a second dose was administered to ensure effective colonic exposure. DSS colitis severity and progression were assessed daily using the DAI, a commonly used measure of disease severity in this model [115]. Moreover, the induction of moderate‐to‐severe colitis was confirmed by the elevated inflammatory biomarkers (lactoferrin and TNF‐α) and histological evidence of severe mucosal damage, including complete loss of the epithelial lining in untreated male animals. Collectively, these findings confirmed the severity of the colitis model. The DAI was calculated based on inflammation associated parameters: body weight loss (percentage of weight loss relative to the initial body weight, where 0, <3%; 1, 3–<10%; 2, 10–<15%; 3, ≥15%), stool consistency (0, normal; 1, sticky and/or moist; 2 mild‐to‐moderate diarrhea; 3 severe diarrhea), and hematochezia (0, no blood; 1, traces of blood in the stool; 2, bloody stool and/or blood around the anus; 3, severe bleeding). On the eighth day, rats were euthanized with intraperitoneal pentobarbital injection (150 mg/kg), colon length was determined, and colon samples were collected for histochemical analysis and TNF‐α quantification. Additionally, stool samples were collected, and lactoferrin concentration was quantified by ELISA following manufacturer's instructions (RTFI00940, AssayGenie).

#### 5.16.1 TNF‐α Quantification

The second most distal 1 cm of the colon was collected for TNF‐α quantification. Colon pieces were lysed with cell lysis buffer (100 µL/5 mg tissue, Bio‐Rad) using a GentleMACS device from Miltenyi Biotec (Miltenyi Biotec, Bergisch Gladbach, Germany), and the concentration of TNF‐α in the lysates was determined by ELISA following the manufacturer's instructions (AEFI01710, AssayGenie).

#### 5.16.2 Histology

To assess the microscopic extent of colitis, the most distal part of the colon (1 cm) was fixed in 4% formalin and subsequently embedded in paraffin. Sections of 5 µm were cut using a rotary microtome (Zeiss, Oberkochen, Germany) and deparaffinized with Histoclear (Chemie Brungschwig, Basel, Switzerland). Sections were rehydrated using a descending series of ethanol (100%–70%) and stained with hematoxylin for 10 min followed by a 2 s differentiating step with 1% HCl and further stained for 10 s with eosin. Sections were dehydrated with an ascending series of ethanol (70%–100%) and mounted with Pertex (HistoLab, Askim, Sweden). Histological scoring was performed by three independent investigators blinded to the type of treatment, and epithelial damage and inflammatory cell infiltration were assessed. A detailed description of the histology scoring system is provided in the Supporting Information.

#### 5.16.3 Histological Quantification of Colonic Morphology

Quantitative histological analysis was performed on the distal colon to assess epithelial integrity [116]. The most distal part of the colon (1 cm) was analyzed for each animal using a systematic uniform random sampling approach. For each sample, three non‐overlapping fields were selected, each consisting of a stitched (patchwork) image generated from 10 high‐magnification micrographs arranged in three rows. The area of preserved luminal epithelial lining was measured within each stitched field, and the measurements from the three fields were pooled. Data were expressed as the percentage of preserved luminal epithelial lining in the 1 cm colon sample and then summarized per treatment group and sex for statistical analysis. This approach ensured unbiased sampling and robust quantification of histological features across the distal colon.

### 5.17 Statistical Analysis

Animals were randomized using a computer‐based random order generator upon arrival, and treatments were randomly assigned within each cage. To ensure objectivity, the evaluating experimenter was blinded to the administered formulations, which were prepared by a second operator. Sample sizes were calculated using G*Power software with a significance threshold of 0.05, a power of 0.90 and an effect size of 0.25 using an F‐test (repeated measures ANOVA, within‐between interaction). In this condition, a sample size of six rats/group was required. Each group included equal numbers of male and female animals, with sex included as a blocking factor in the statistical analysis. Because disease severity was sex‐dependent, with males developing more severe disease, sexes were analyzed separately in Figure 5 (panels C, L, and N). Results are presented as mean ± SD or mean ± SEM. Multiple groups were compared using parametric tests (one‐way ANOVA) with Tukey's post‐hoc correction for multiple comparisons. However, in the case of PD Studies (DAI; Figure 5, Panels A, K, and L), a linear mixed‐effects model (LME) was applied. The LME was selected to account for the repeated measurements taken from individual animals over time (random effect) and to handle the unbalanced nature of the dataset. Fixed effects included Group, Time, and their interaction. Statistical details for each in vivo experiment (including sample size, handling of outliers, and strategies for group comparisons) are also provided in the figure captions.

## Author Contributions

**Rafaela Gazzi**: writing the original draft, writing the review & editing, visualization, validation, methodology, investigation, formal analysis, data curation, and conceptualization. **Tiziana Cremona**: writing the original draft, writing the review & editing, validation, methodology, investigation, formal analysis, data curation, and conceptualization. **Reto Czekalla**: investigation and formal analysis. **Emily Campbell**: writing the review & editing, investigation, and formal analysis. **Marlene Schwarzfischer**: writing the review & editing, investigation, and formal analysis. **Doris Pöhlmann**: formal analysis (histology). **Vanessa Dobernig**: formal analysis (histology). **Gerhard Rogler**: writing the review & editing and resources. **Michael Scharl**: writing the review & editing, resources. **Alessandra Bergadano**: writing the review & editing. **Raffaele Mezzenga**: writing the review & editing, formal analysis (saxs), and resources. **Martin Kuentz**: writing the review & editing and resources. **Paola Luciani**: writing the review & editing, resources, supervision, and funding acquisition. **Simone Aleandri**: writing the original draft, writing the review & editing, methodology, investigation, formal analysis, data curation, conceptualization, supervision, funding acquisition, and project administration.

## Funding

The authors gratefully acknowledge the Swiss National Science Foundation for the financial support (Grant Nr. 10.003.897).

## Conflicts of Interest

The authors declare the following financial interests/personal relationships that may be considered potential competing interests: No private study sponsors were involved in the study design, data collection, or interpretation of the data presented in this manuscript. Paola Luciani and Simone Aleandri have received research grants from PPM Services S.A.

## Supporting information




**Supporting File**: advs78234‐sup‐0001‐SuppMat.docx.

## Data Availability

The data that support the findings of this study are available from the corresponding author upon reasonable request.
