# Supplementary material for: Advancing Biotherapeutic Treatment of Ulcerative Colitis Using Lipid Mesophases: Formulation Design and Preclinical Validation
Source: Adv Sci (Weinh). 2026 Oct 11:e78234. Online ahead of print. doi: 10.1002/advs.78234 (PMC13655260; doi:10.1002/advs.78234)
Supplement: Supplementary file 1 [file ADVS-9999-e78234-s001.docx]

Supporting Information

Advancing Biotherapeutic Treatment of Ulcerative Colitis Using Lipid Mesophases: Formulation Design and Preclinical Validation.

Rafaela Gazzi, Tiziana Cremona, Reto Czekalla, Emily Campbell, Marlene Schwarzfischer, Doris Pöhlmann, Vanessa Dobernig, Gerhard Rogler, Michael Scharl, Alessandra Bergadano, Raffaele Mezzenga, Martin Kuentz, Paola Luciani, Simone Aleandri*

*Corresponding author. Correspondence to be sent to: [simone.aleandri@unibe.ch](mailto:simone.aleandri@unibe.ch)

**Supporting Text**

**Materials**

S80 (with 75% polyenylphosphatidylcholine) and Lipoid E PC S (phosphatidylcholine from egg yolk) were obtained from Lipoid GmbH (Ludwigshafen, Germany). Our source of monolinolein was Dimodan U/J, which was kindly donated by Danisco (Copenhagen, Denmark). Dimodan U/J is an industrial food-grade quality analogue of MLO composed of a mixture of monoglyceride derived from oleic fatty acid with a minimum monoglyceride content of 90%. Sucrose stearate was purchased from abcr (Karlsruhe, Germany). Vitamin E (Ph. Eur. Quality), lipase from Candida rugosa, Trizma® maleate, calcium chloride dihydrate (≥99%), pancreatin (from porcine pancreas, 8xUSP specifications) and maleic acid were purchased from Sigma-Aldrich Chemie GmbH (Buchs, Switzerland). Phosphate buffer saline was obtained from Capricorn Scientific GmbH (Ebsdorfergrund, Germany). Disodium phosphate, embedding cassettes and sponges for histology and formaldehyde solution (37%) were obtained from Carl Roth (Karlsruhe, Germany). Sodium dihydrogen phosphate dihydrate was obtained from Hänseler AG (Herisau, Switzerland). Sodium taurodeoxycholate was obtained from Prodotti Chimici e Alimentari S.p.A. (Basaluzzo, Italy). Barium sulphate (Puratronic, 99.998%) was purchased from Thermo Fisher Scientific (Switzerland). Hydrochloric acid 37% pure (HCl) was supplied by Dr. Grogg Chemie (Switzerland). Eudragit® FS30D was kindly donated by Evonik (Rheinfelden, Germany). Simulated digestive fluids were purchased from Biorelevant Ltd. (London, UK). Infliximab (IFX, Remicade) was purchased from the Inselspital (Bern, Switzerland). Infliximab (HUMB00005), rat lactoferrin (RTFI00940) and rat TNF-α (AEFI01710) ELISA kits were obtained from AssayGenie (Dublin, Ireland). 1,1′-dioctadecyl-3,3,3′,3′-tetramethylindodicarbocyanine, 4chlorobenzenesulfonate salt (DiD) was obtained from Invitrogen (Carlsbad, USA). Gelatine capsules size 4 were purchased from Interdelta (Switzerland), and gelatine capsules size 9 were purchased from Torpac (Heerlen, Netherlands). Dextran sodium sulphate (DSS, 40kDa) was obtained from TdB Labs (Uppsala, Sweden). Cell lysis kit was purchased from Bio-Rad (Switzerland).

**Preparation of LMPs**

*S80/VitE LMP*

S80 (80%) and vitamin E (12%) were co-dissolved in methanol into a glass vial, and the organic solvent was removed under reduced pressure (0.22 mbar for 24 h). Water (20%) was incorporated into the dried lipid, and the mixture was manually mixed with a spatula and centrifuged for 10 min at 5’000 x g, which was repeated six times to obtain a uniform and homogeneous gel. The LMP was allowed to equilibrate for 48 h in the dark at room temperature.

*Preparation of MLO/SE LMP*

Monolinolein (MLO) and sucrose stearate (SE) mixture (50% w/w) was transferred to a syringe (Fisher Scientific, Pittsburgh, US), and the aqueous solution (PBS or IFX solution, 50%) was added to a second syringe. Both syringes were attached to a syringe connector (Combifix^®^ Adapter Luer-Lock female/female), and the LMP was hydrated by transferring the contents of one syringe to the other for three min. The final gel was equilibrated in the syringe for 10 min.

**Preparation of infliximab solution and drug quantification**

Remicade^®^ vials were resuspended in ultrapure water to a concentration of 30 mg/mL and used to produce rectal gels. For comparison in vivo, the solution was diluted to 15 mg/mL and administered as an enema. The 30 mg/mL IFX solution was concentrated using a 50’000 MWCO Amicon® Ultra centrifugal filter to 100 mg/mL to print the core of the human printlet, and to 180 mg/mL to print the core of the rat printlet. The concentration of IFX in preliminary release studies in PBS and simulated intestinal fluids was determined by HPLC as described below. The concentration of IFX in confirmatory release studies in simulated intestinal fluids and in vivo experiments was determined by ELISA (HUMB00005, AssayGenie) following manufacturer’s instructions.

**HPLC method for infliximab quantification**

IFX concentrations were determined by reversed-phase high-performance liquid chromatography (RP-HPLC) using a Biozen dSEC 2 (00H-4788-K0, Phenomenex, USA). The HPLC method was adopted from the European Pharmacopoeia, with a flow rate of 1 mm/s, column temperature of 25°C, detection wavelength of 214 nm, injection volume of 10 µL, and run time of 20 min. The sampler was kept at 10°C. The mobile phase was composed of 6.5 mM NaH_2_PO_4_, 13.5 mM Na_2_HPO_4_, and 150 mM NaCl.

**Circular dichroism**

To determine if the concentration of IFX affects its structure, circular dichroism measurements were performed on IFX solutions with concentrations of 30, 100, 180 mg/mL. A J-75 detector coupled with a PS150J power unit (JASCO, Germany) was used. The resulting spectra were then compared to IFX spectra from the literature to determine if the high concentration has an influence on the structure. The solutions were diluted to 0.05 mg/mL for successful measurements. Cuvettes with a 1 cm path length were used for measurements between 205 and 250 nm at room temperature.

**Design of Experiments**

The optimal printing parameters were determined using a full factorial DoE generated by Minitab^®^ 18.1 software. Extruder temperature (three levels: 30, 35, and 40°C), speed (three levels: 3, 5, and 7 mm/s), and pressure (four levels: 80, 110, 150, and 200 kPa) were selected as factors. In total, 36 runs were performed for each of the six LMPs composed of different percentages of MLO and SE, and a printability score (from 0 to 5, where 0 was not possible to print, and 5 the optimal printlet shape and consistency) was used as response. A scoring guide for printability is shown in Figure S1. From the printability scores, a model was calculated to determine the influence of factors on the results, and which factors have statistically significant effects on the response (p ≤ 0.10).

**Lipolysis assay of the 3D-printed oral dosage form**

A micellar solution simulating fasted-state intestinal conditions was prepared by dissolving phosphatidylcholine (1.25 mM) and sodium taurodeoxycholate (5 mM) in a digestion buffer (50 mM Trizma^®^ maleate, 150 mM NaCl, 5 mM CaCl₂·2H₂O, pH 7.5). The mixture was stirred for 12 h at 5°C (450 rpm). A pancreatin extract was freshly prepared by dispersing porcine pancreatin powder (1 g/5 mL digestion buffer), stirring for 15 min at 5°C, and centrifuging (15 min, 1600 x g, 5°C). The supernatant pH was adjusted to 7.5 and kept on ice until further use. The lipolysis experiment was conducted by adding one 3D-printed tablet to 60 mL of the micellar medium in a thermostated vessel (37 ± 0.5°C) under stirring (700 rpm). After 10 min of equilibration, digestion was initiated by adding pancreatin extract (final nominal lipase activity: 1000 tributyrin units/mL). Free FA released during digestion were titrated automatically with 1 M NaOH to maintain a pH of 7.5 using a pH-stat apparatus (842 Titrando and 800 Dosino, Metrohm AG, Switzerland). Lipolysis proceeded for 270 min. To correct for unionized free FA, back-titration was performed by rapidly increasing the pH to 9 with 1 M NaOH at the end of digestion. A blank digestion (without formulation) was also performed, and its NaOH consumption was subtracted to obtain the corrected free FA value (FAtitr). The correction factor was calculated according to the formula:

Correction factor = (FAtitr (direct titration) + FAtitr (back titration)) / FAtitr (direct titration)

Where FAtitr (direct titration) is the amount of FA titrated after the 270 min digestion period.

A correction factor was calculated for both the lipolysis of the 3D-printed tablet and pure digestion medium. The amount of free FA released from the pure digestion medium was then subtracted from the total FA measured in the presence of the tablet.

**In vivo experiments**

Rats were housed under specific pathogen-free conditions in compliance with the Federation of European Laboratory Animal Science Associations (FELASA) guidelines. Animals were kept in groups of up to three in autoclaved individually ventilated cages (Blue Line, 1500 cm^2^, Tecniplast, Italy) containing aspen wood bedding (J. Rettenmeier & Söhne GmbH, Germany) and cotton nestlets that were used for nesting. Environmental enrichment was provided in the form of a black PVC tunnel (Plexx, Netherlands) and an aspen wood stick (LAB & VET Service GmbH, Austria). Autoclaved tap water and irradiated rodent chow (Mouse and Rat Maintenance 3432; Granovit, Switzerland) were provided ad libitum. Housing conditions were maintained under a 12:12 h light-dark cycle, at a room temperature of 22 ± 2°C and a relative humidity of 45-65%. Animals were allowed a one-week acclimatization period and were regularly handled by trained personnel for gentling and habituation to the experimental procedures.

**Histology scoring**

Epithelial damage: intact epithelium = 0; focal goblet cell depletion in limited areas and/or mild irregularity in crypt spacing = 1; moderate goblet cell loss across larger areas and/or pronounced irregular crypt spacing = 2; extensive loss of goblet cells and crypt structures = 3; near complete or complete loss of crypts over large areas = 4.

Infiltration: no infiltrate = 0; mild infiltration around crypt basis = 1; moderate infiltration extending into the submucosa = 2; extensive infiltration throughout the submucosa and oedema = 3; dense infiltration into both submucosa and muscularis externa, prominent oedema and tissue disruption = 4.

The total histology score represents the sum of the epithelium damage score and the infiltration score. Images were taken using a Zeiss Axio Imager.Z2 microscope (Zeiss), equipped with an AxioCam HRc (Zeiss, Jena, Germany) camera and ZEN imaging software (Zeiss, Germany).

**Supporting Tables**

**Table S1.** **Printability scores for DoE runs using LMPs with lipid mixture containing 0-40% sucrose stearate.**

| Factors | | | Printability score of tested LMPs | | | | | |
| --- | --- | --- | --- | --- | --- | --- | --- | --- |
| Pressure (kPa) | Speed (mm/s) | Temperature (°C) | MLO_100_SE_0_ | MLO_80_SE_20_ | MLO_75_SE_25_ | MLO_70_SE_30_ | MLO_65_SE_35_ | MLO_60_SE_40_ |
| 150 | 7 | 40 | 0 | 1 | 1 | 1 | 1 | 1 |
| 150 | 5 | 40 | 0 | 1 | 1 | 1 | 1 | 1 |
| 200 | 5 | 40 | 0 | 1 | 1 | 1 | 1 | 1 |
| 200 | 5 | 35 | 0 | 2 | 1 | 1 | 1 | 1 |
| 110 | 5 | 30 | 0 | 1 | 1 | 4 | 1 | 1 |
| 80 | 5 | 35 | 0 | 1 | 1 | 2 | 4 | 2 |
| 110 | 5 | 40 | 0 | 1 | 1 | 2 | 1 | 1 |
| 200 | 7 | 30 | 0 | 1 | 1 | 1 | 1 | 1 |
| 150 | 3 | 30 | 0 | 1 | 3 | 1 | 1 | 1 |
| 150 | 7 | 30 | 0 | 1 | 1 | 1 | 1 | 1 |
| 110 | 7 | 35 | 0 | 1 | 1 | 2 | 2 | 1 |
| 80 | 5 | 40 | 0 | 1 | 1 | 1 | 1 | 3 |
| 200 | 7 | 40 | 0 | 2 | 1 | 1 | 1 | 1 |
| 80 | 3 | 35 | 0 | 1 | 1 | 2 | 4 | 1 |
| 200 | 3 | 30 | 1 | 1 | 1 | 1 | 1 | 1 |
| 110 | 3 | 30 | 0 | 1 | 1 | 3 | 1 | 1 |
| 110 | 3 | 40 | 0 | 1 | 1 | 4 | 1 | 1 |
| 200 | 5 | 30 | 0 | 1 | 1 | 1 | 1 | 1 |
| 80 | 7 | 35 | 0 | 1 | 1 | 1 | 2 | 2 |
| 200 | 7 | 35 | 0 | 2 | 1 | 1 | 1 | 1 |
| 80 | 5 | 30 | 0 | 1 | 1 | 1 | 4 | 1 |
| 200 | 3 | 40 | 1 | 1 | 1 | 1 | 1 | 1 |
| 80 | 3 | 40 | 0 | 1 | 1 | 1 | 3 | 1 |
| 110 | 5 | 35 | 0 | 1 | 1 | 3 | 1 | 1 |
| 80 | 3 | 30 | 0 | 1 | 1 | 1 | 5 | 1 |
| 110 | 7 | 40 | 0 | 1 | 1 | 1 | 2 | 1 |
| 110 | 3 | 35 | 0 | 1 | 1 | 5 | 1 | 1 |
| 110 | 7 | 30 | 0 | 1 | 1 | 3 | 1 | 1 |
| 150 | 3 | 35 | 0 | 1 | 2 | 1 | 1 | 1 |
| 80 | 7 | 40 | 0 | 1 | 1 | 1 | 1 | 4 |
| 150 | 5 | 35 | 0 | 1 | 2 | 1 | 1 | 1 |
| 150 | 5 | 30 | 0 | 1 | 2 | 1 | 1 | 1 |
| 80 | 7 | 30 | 0 | 1 | 1 | 1 | 4 | 2 |
| 200 | 3 | 35 | 1 | 1 | 1 | 1 | 1 | 1 |
| 150 | 7 | 35 | 0 | 1 | 1 | 1 | 1 | 1 |
| 150 | 3 | 40 | 0 | 1 | 1 | 1 | 1 | 1 |

**Table S2.** **Ratio of lipid (MLO) and sucrose stearate (SE) in the lipid mixture and the corresponding LMP nomenclature.**

| LMP Nomenclature | Monolinolein (%) | SE (%) |
| --- | --- | --- |
| MLO_100_SE_0_ | 100 | 0 |
| MLO_80_SE_20_ | 80 | 20 |
| MLO_75_SE_25_ | 75 | 25 |
| MLO_70_SE_30_ | 70 | 30 |
| MLO_65_SE_35_ | 65 | 35 |
| MLO_60_SE_40_ | 60 | 40 |

**Supporting Figures**

**Figure S1.** **Printability scoring guide for the DoE runs.** Scores 1-4 correspond to printing conditions that produce excessive (top) or insufficient (bottom) ink extrusion. Score 5 indicates an optimal 3D-printed core. Score 0 is assigned when no ink is extruded from the nozzle (not shown).

**Figure S2.** **Main effects and interaction plots for printability of LMPs with sucrose stearate 20-40%.** Main effects plots for the printability of **(A)** MLO_80_SE_20_ and **(B)** MLO_75_SE_25_. **(C)** Main effects and **(D)** interaction plots (pressure × speed) for the printability of MLO_70_SE_30._ **(E)** Main effects and **(F)** interaction plots (left: pressure × speed, right: pressure × temperature) for printability of MLO_65_SE_35._ **(G)** Main effects and **(H)** interaction (left: pressure × speed, right: pressure × temperature) plots for printability of MLO_60_SE_40._

**Figure S3.** **Pareto charts of the standardized effects on the printability of LMPs with sucrose stearate at 20-40%.** **(A)** MLO_80_SE_20_, **(B)** MLO_75_SE_25_, **(C)** MLO_65_SE_35_ and **(D)** MLO_60_SE_40_.

**Figure S4.** **SAXS spectra of MLO_70_SE_30_ hydrated with 45% or 50% PBS.** Both spectra show Bragg peaks with positions in the ratio √2:√3:√4:√6:√8:√9, characteristic of an inverse bicontinuous cubic phase with Pn3m symmetry. Both LMPs present the first Bragg peak (√2) at q 0.07 nm^-1^. The LMP is at maximum hydration as the first Bragg peak (√2) does not change its position while increasing the hydration percentage hydration. In contrast, MLO_100_SE_0_ reaches its maximum hydration with 40% of PBS and its first Bragg peak is at q > 0.15 nm^-1^ (see Fig. 1, panel K upper spectra).

**Figure S5. SAXS spectra of LMPs composed of 50% lipid mixture of MLO and sucrose stearate (SE) and 50% PBS.** Top spectra: lipid mixture composed of 45% MLO and 55% SE. Middle spectra: Lipid mixture composed of 50% MLO and 50% SE. Bottom spectra: Lipid mixture composed of 55% MLO and 45% SE. These percentages of SE lead to LMPs with unknown crystalline domains.

**Figure S6.** **Technical drawings of the designed 3D-printed oral dosage forms.** **(A)** Tablet designed for human use. **(B)** Scaled-down tablet for in vivo experiments in rats.

**Figure S7.** **Far-UV circular dichroism spectra of infliximab solutions at 30, 100, and 180 mg/mL.** A strong negative band near 217 nm, indicative of the β-sheet secondary structure, was consistent across all concentrations.

**Figure S8. Free fatty acids (FA) originated from the digestion of InflixiPrint or its individual components.** InflixiPrint, S80/VitE LMP in the shell and MLO_70_SE_30_ LMP in the core. Results are shown as mean ± SD (n=2).

**Figure S9.** **Representative images of oral dosage forms after 2 h of incubation in HCl (0.1 M).** **(A)** InflixiCaps and **(B)** InflixiPrint.

**Figure S10.** **Total fluorescence counts of colon tissues.** Untreated colon tissues or colon tissues at 8 and 24 h after administration of rectal formulations (InflixiGel and enema) loaded with DiD dye. Results are shown as single point values ± SD (n=3). Statistical significance was evaluated using two-way ANOVA with Tukey’s post hoc analysis. The adjusted p-values are reported above the comparison.

**Figure S11.** **Colonic concentration of IFX after rectal administration of InflixiGel or enema to healthy rats.** Results are shown as single point values ± SD (n=3). Statistical significance was evaluated using one-way ANOVA with Tukey’s post hoc analysis. The adjusted p-values are reported above the comparison.

**Figure S12.** **Body weight loss of untreated rats and rats treated with InflixiGel and InflixiCaps during DSS cycle.** **(A)** 4% DSS (w/v) cycle and **(B)** 6% DSS (w/v) cycle. Results are shown as mean ± SEM (n=6).

**Figure S13.** **Disease activity index (DAI) of untreated males and females during DSS cycle (4% w/v).** Results are shown as mean ± SEM (n=3). Statistical significance was evaluated using linear mixed-effects, and the adjusted p-value corresponds to the comparison on the 7^th^ day.

**Figure S14.** **Colon length of naïve and untreated males and females after DSS cycle (4% w/v).** Results are shown as single point values ± SD. Statistical significance was evaluated using one-way ANOVA with Tukey’s post hoc analysis. The adjusted p-values are reported above the comparison.

**Figure S15.** **Disease activity index (DAI) of untreated males and females during DSS cycle (6% w/v).** Results are shown as mean ± SEM (n=3). Statistical significance was evaluated using linear mixed-effects, and the adjusted p-value corresponds to the comparison on the 7^th^ day.

**Figure S16.** **Colon length of naïve and untreated males and females after DSS cycle (6% w/v).** Results are shown as single point values ± SD. Statistical significance was evaluated using one-way ANOVA with Tukey’s post hoc analysis. The adjusted p-values are reported above the comparison.

**Figure S17.** **Behavior of InflixiCaps and PowderCaps in solution.** Representative images of InflixiCaps (left) and PowderCaps (right). Capsules are indicated by arrows.

**Figure S18. Disease-related endpoints of untreated rats and rats treated with subcutaneous injection of IFX after DSS cycle (6% w/v). (A)** Disease activity index (DAI), **(B)** colon length and **(C)** fecal lactoferrin concentration. In panel A results are shown as mean ± SEM (n=3) and statistical significance was evaluated using linear mixed-effects. In panels B and C results are shown as single point values ± SD. Statistical significance was evaluated using nonparametric Mann-Whitney test and the adjusted p-values are reported above the comparison.

**Figure S19. Histological analysis of colons from DSS-exposed rats (6% w/v) after systemic IFX treatment.** Representative histology images of **(A)** untreated rats and **(B)** rats treated with subcutaneous injection of IFX after DSS cycle (6% w/v), and **(C)** their histology score. Results are shown as single point values ± SD. Statistical significance was evaluated using nonparametric Mann-Whitney test and the adjusted p-value is reported above the comparison. ± SD.
